# The crucial impact of iron deficiency definition for the course of precapillary pulmonary hypertension

**DOI:** 10.1371/journal.pone.0203396

**Published:** 2018-08-30

**Authors:** Thomas Sonnweber, Manfred Nairz, Igor Theurl, Verena Petzer, Piotr Tymoszuk, David Haschka, Eva Rieger, Birgit Kaessmann, Miriam Deri, Kathrin Watzinger, Regina Steringer-Mascherbauer, Ivan Tancevski, Günter Weiss, Judith Löffler-Ragg

**Affiliations:** 1 Department of Internal Medicine II, Medical University Innsbruck, Innsbruck, Austria; 2 Department of Internal Medicine II, Elisabethinen Hospital, Linz, Austria; 3 Christian Doppler Laboratory for Iron Metabolism and Anemia Research, Medical University Innsbruck, Innsbruck, Austria; National Institute of Child Health and Human Development, UNITED STATES

## Abstract

Imbalances of iron homeostasis are associated with an adverse clinical outcome of pulmonary hypertension (PH). Herein, we aimed to analyze the impact of iron deficiency (ID) in a real-life PH patient cohort according to different currently used ID definitions. In a retrospective study including 153 precapillary PH patients followed over a mean period of five years, iron deficiency was assessed according to five clinical definitions used in previous trials. The impact of ID on clinical, hematological and hemodynamic parameters was investigated. Depending on the different cutoff levels for serum ferritin and transferrin saturation, currently used ID definitions indicated a prevalence of either true or functional ID in 11 to 75 percent of PH patients. A good diagnostic accuracy was achieved by using the sTFRF/log ferritin (sTFRF) index, which identified 33 to 42 percent of PH patients as being iron deficient. The sTFRF index had the best prediction for the association between ID and clinical outcome. Iron deficient patients with precapillary PH had a significantly higher mortality as compared to non-iron deficiency subjects, which was true for both, PH patients with and without anemia. Although levels of the iron hormone hepcidin were rather affected by ID than by inflammation, they were not associated with the clinical course or mortality of PH subjects. To conclude, ID had a significant impact on the clinical course of precapillary PH patients. The appropriate use of robust biomarkers to define ID is a prerequisite to further evaluate the role of ID and the potential benefit of iron supplementation in precapillary PH patients.

## Introduction

Iron is a key element for numerous vital processes, including hemoglobin synthesis, mitochondrial respiration, immune-surveillance, cellular proliferation and metabolic activity [1–3]. Contrary, excess iron catalyzes radical formation and leads to cellular damage [4]. On the systemic level, dietary iron absorption and iron re-distribution from macrophages are controlled by the hormone hepcidin [5–7], which binds to the cellular iron export protein ferroportin, thereby controlling cellular iron egress. A complex network of signaling cascades control hepcidin expression, and its circulating concentrations are mainly increased by iron loading and inflammation, whereas they are reduced by iron deficiency (ID), hypoxia, erythropoietic activity or hormones [8].

Recently, imbalances in iron homeostasis have been recognized in patients with chronic heart failure and pulmonary arterial hypertension (PAH) [9–11]. Iron supplementation in patients suffering from ID with and without anemia and chronic heart failure resulted in beneficial effects [9, 12]. Of note, the definition of ID greatly varied in recent trials [9–12] and ID is caused by completely different pathophysiologies resulting in contrasting body iron distribution. First, there is true or absolute ID, arising from insufficient iron absorption or mostly from iron losses as a consequence of urogenital or gastrointestinal bleeding episodes [13]. Second, inflammatory processes result in functional iron deficiency as a consequence of inflammation and hepcidin driven iron retention in macrophages [1].

In absolute ID, ferritin levels, a surrogate for iron storage, are low indicating a scarcity of iron in the circulation. If absolute ID persists, the lack of iron for hemoglobin synthesis in the bone marrow leads to the development of ID anemia (IDA) [13]. In functional ID iron is retained in the mononuclear phagocyte system, as characterized by increased ferritin and hepcidin levels, whereas the metal is not sufficiently accessible for erythropoiesis, which results in the development of anemia of chronic disease (ACD) [1, 8, 14].

PH is characterized by complex vascular remodeling and occlusion of pulmonary vessels resulting in right heart hypertrophy and failure [15, 16]. Its pathogenesis, although incompletely understood, involves the complex interplay of genetic factors, inflammatory triggers, hypoxia and metabolic changes. ID is frequently found in patients with idiopathic pulmonary hypertension (IPAH) and correlates with both, disease severity and clinical outcome [10, 11]. Consequently, current ESC/ERS guidelines suggest a regular monitoring of iron status in PH patients and eventual iron supplementation [17].

Several crosslinks between iron metabolism and the pathophysiology of PH exist. First, mutations in the TGFβ receptor family, most notably bone morphogenetic protein receptor II (BMPRII), are found in several PAH patients [18]. Second, the BMP/SMAD cascade is a central regulator of hepcidin production [19] and hepcidin expression can be induced in PH by inflammatory stimuli either systemically or loco-regionally in tissue macrophages [6, 7, 20]. Third, PH patients exhibit disturbances of mitochondrial function, which might reflect reduced iron availability, as iron is central for numerous mitochondrial enzymes [21, 22]. Fourth, hypoxia links iron metabolism to PH, because hypoxia-inducible factors (HIFs), growth factors, such as PDGF-BB, and the nitric oxide (NO) pathway are implicated in both, modulation of iron homeostasis and vascular remodeling in PAH [11, 23–25]. In this context, two studies directly linked iron regulatory protein 1 (IRP1) and von-Hippel-Lindau (VHL) factor dependent HIF-2α regulation to the emergence of pulmonary hypertension [26, 27], and ID is associated with exaggerated pulmonary arterial pressure following hypoxic challenge [28]. Thus, it appears obvious that disturbances of iron homeostasis affect PH. Importantly, no systemic evaluation of the causes of iron deficiency (e.g. absolute versus functional iron deficiency) in PH patients has been performed thus far, and ongoing clinical trials use different criteria to define ID (NCT01446848, NCT01288651, NCT01447628, and NCT03371173). This incoherence of ID definition is of concern, as it affects the choice and efficacy of iron modifying treatments in PH, depending on the underlying pathophysiology and type of iron-misdistribution. Although retrospective epidemiology studies suggest an association of ID with mortality and exercise capacity of PH patients, it is not clear whether this applies to both, absolute and functional ID, or whether disturbances of iron homeostasis emerge during the course of the disease or are relevant contributors to PH itself.

In the presented study, we conducted a retrospective analysis in a heterogeneous “real life” cohort of PH patients, addressing the prevalence and type of ID and anemia, respectively. We compared five different definitions of ID, previously used in clinical studies, in respect to their sensitivity and specificity and evaluated the impact of ID on the clinical course of PH.

## Materials and methods

### Patient data analyses

Data from 153 adult patients (age range 19 to 90 years) diagnosed with precapillary PH were retrospectively analyzed. Patients were followed up for a mean observation period of five years. We evaluated laboratory tests, blood gas analyses, clinical assessment (including six-minute walking distance (SMWD) and WHO functional class (WHOFc)), right heart catheter results and echocardiographic findings. Mortality risk was evaluated according to the current ESC/ERS guidelines [17]. Blood sampling was performed via routine peripheral vein puncture, and we obtained blood gas analysis via punctuation of the hyper-perfused earlobe following Finalgon® (Sanofi-Aventis, Frankfurt am Main, Germany) application. Patients provided written informed consent prior participation and consented to use their medical records and samples for research purposes. All samples and data were fully anonymized. The sampling, storage of additional blood samples and retrospective analysis of patient samples and data was reviewed and approved by the ethical committee of the Medical University of Innsbruck in accordance with the Declaration of Helsinki (approval numbers: AM2544, 239/4.12 and 273/5.7, AN2017-0009369/4.15).

Determination of hepcidin 25 (DRG Instruments GmbH, Germany) and IL6 (both from BD Biosciences, San Diego, CA, USA) was done by enzyme-linked immune absorbent assay (ELISA). All other laboratory parameters were determined by standard automated methods in the certified central laboratories of the two hospitals participating in this study.

### Definition of anemia and iron deficiency

Anemia was defined by hemoglobin levels below 120g/L for women and below 130g/L for men. Anemia was categorized as iron deficiency anemia (IDA, anemia with a serum ferritin below 30μg/L), anemia of chronic disease (ACD; anemia and a serum ferritin above 100μg/L, or serum ferritin between 30–100μg/L and a sTFR-Ferritin (sTFRF) index, as calculated by the ratio of sTfR/log ferritin, below 1) or combination of both (ACD+IDA, serum ferritin between 30–100μg/L and sTFRF index above 2).

We used five different definitions of ID, described in literature [1, 9, 29–32]. Based on serum ferritin and transferrin saturation (TSAT), we implemented a strict (ID defined as serum ferritin <30μg/L and TSAT <16%, referred to as ID1) and a liberal definition of ID (serum ferritin <100μg/L and TSAT <20%, referred to as ID2). ID3 considers ID criteria of several cardiology studies (ferritin <100μg/L or ferritin 100–299μg/L and TSAT <20%) [9, 30]. Further, we used sTFR (ID defined by sTFR >4.5 for women and >5.0 for men, referred to as ID4) and the sTFRF index with correction for CRP levels (ID defined by sTFRF index >3.2 if CRP <0.5 mg/dL or sTFRF index >2 if CRP >0.5 mg/dL, referred to as ID5).

### Statistical analysis

Data analysis was carried out with statistical analysis software package (IBM SPSS Statistics version 24.0; IBM, New York, USA). A descriptive data evaluation including tests for homoscedasticity, random distribution, and normal distribution was performed. Parametric Student`s t-test and repeated measures analysis of variance (ANOVA) or non-parametric Mann-Whitney-U test and Kruskal-Wallis test were applied, as appropriate. Trends over time analyses were conducted with paired Student`s t-test, Wilcoxon test or Nemar`s test, as appropriate. Pearson (parametric data) or Spearman-rho (non-parametric data) test were used to analyze for significant associations among various parameters. We assessed time-dependent mortality during the observation period using log-rank (Mantel-Cox), Breslow (generalized Wilcoxon) and Tarone-Ware test. Sensitivity and specificity of differential definitions of iron deficiency were evaluated with receiver operating characteristic (ROC) analysis.

## Results

### Patients' characteristics at first consultation and follow up

At the first consultation, a comprehensive evaluation including assessment of comorbidities and treatment modalities, clinical performance status, laboratory tests, echocardiography and right heart catheterization parameters, blood gas analysis, pulmonary function testing and clinical risk score assessment was performed (Table 1, S1 and S2 Tables). The majority of patients had type 1 pulmonary arterial hypertension (61.6%) or inoperable type 4 chronic thromboembolic pulmonary hypertension (CTEPH, 19.9%). Group 1 PH patients were mostly classified as idiopathic PAH (iPAH), and also included a subgroup of 15 patients with connective tissue disease associated precapillary PH (9 patients with CREST (calcinosis cutis—Raynaud phenomenon—esophageal dysmotility—sclerodactyly—telangiectasia) syndrome, 3 patients with systemic sclerosis (SSc), 2 patients with systemic lupus erythematosus (SLE) and 1 patient with mixed connective tissue disease). Individuals with operable CTEPH, cardiac shunts or hemolytic anemias were not included in the study. Patients mainly presented with WHOFc II (35.9%) and WHOFc III (52.9%), with a mean SMWD of 376.3 (±125.1) meters at first consultation. The clinical mortality risk-assessment score [17], categorized 41.6% of patients at low, 46.5% at intermediate and 11.9% at high mortality risk, respectively (S1 Table). Comorbidities, symptoms, and medications, which could impact on iron homeostasis were present in 85.5% of PH patients (S2 Table).

**Table 1:** Patients' characteristics at first consultation (N = 153).

**clinical parameters**	**mean**	**±**	**SD**	**reference range**	**right heart catheterization**	**mean**	**±**	**SD**	**reference range**
age (years)	67.5	±	14.0	n.a.	PAPm (mmHg)	40.5	±	15.9	≤20.0
BMI (kg/m^2^)	26.7	±	5.7	18.5–24.9	RAPm (mmHg)	11.0	±	5.1	≤7.0
SMWD (meter)	376.3	±	125.1	>400.0	Cardiac index (L/min/m^2^)	2.4	±	0.6	2.5–4.0
					PCWP (mmHG)	15.5	±	6.9	≤12.0
**gender**	**N (%)**				PVR (dynxsxcm-5)	559.0	±	408.7	<250.0
female	90 (58.8)				SvO2 (%)	65.3	±	8.0	68.0–78.0
male	63 (41.2)				TPG (mmHG)	24.4	±	15.7	<12.0
**laboratory parameters**	**mean**	**±**	**SD**	**reference range**	**echocardiography**	**mean**	**±**	**SD**	
hemoglobin (g/L)	140.1	±	21.6	120.0–157.0	sPAP (mmHg)	59.9	±	20.7	<30.0
RDW (%)	15.0	±	2.1	11.0–16.0	TAPSE (mm)	20.9	±	5.6	>16.0
MCV (fL)	89.3	±	6.1	77.0–96.0	RVEDD (mm)	37.5	±	8.3	<30.0
MCH (pg)	29.8	±	2.4	27.0–32.0	LVEF (%)	54.9	±	11.0	≥55.0
serum iron (μmol/L)	16.8	±	8.0	5.8–34.5					
transferrin (mg/dL)	267.4	±	54.9	200.0–360.0	**arterial blood gas analysis**	**mean**	**±**	**SD**	
transferrin saturation (%)	25.5	±	14.2	16.0–45.0	pO2 (mmHg)	68.8	±	13.8	80.0–100.0
ferritin (μg/L)	144.9	±	142.0	30.0–400.0	pCO2 (mmHg)	35.9	±	6.3	35.0–45.0
NTproBNP (ng/l)	1977	±	4365	0–486	AaDO2 (mmHg)	31.5	±	12.8	0.0–20.0
CRP (mg/dL)	0.8	±	1.5	0.0–0.5					
GFR mL/min/1.73m^2^)	59.6	±	18.0	≥60.0	**pulmonary function tests**	**mean**	**±**	**SD**	
uric acid (mg/dL)	6.9	±	2.1	3.6–7.0	DLCO (%)	67.6	±	24.9	80.0–100.0
creatinine (mg/dL)	1.1	±	0.8	0.67–1.17	KCO (%)	82.8	±	29.3	80.0–100.0

Data are represented as mean ± 1 standard deviation (SD); N depicts number of valid data for retrospective analysis; abbreviations: BMI, body mass index; SMWD, six minute walking distance; RDW, red blood cell distribution width; MCV, mean corpuscular volume; MCH, mean corpuscular hemoglobin; NTproBNP, N-terminal pro-B-type natriuretic peptide; CRP, C reactive protein; GFR, glomerular filtration rate; PAPm, mean pulmonary arterial pressure; RAPm, mean right atrial pressure; PCWP, pulmonary capillary wedge pressure; PVR, pulmonary vascular resistance; SvO2, mixed venous saturation; TPG, transpulmonary pressure gradient (PAPm-PCWP); sPAP, systolic pulmonary arterial pressure; TAPSE, tricuspid annular plane systolic excursion; RVEDD, right ventricular end diastolic diameter; LVEF, left ventricular ejection fraction; pO2, arterial partial pressure of oxygen; pCO2, arterial partial pressure of carbon dioxide; AaDO2, alveolar-arterial oxygen difference; DLCO, diffusing capacity for carbon monoxide, depicted as percentage of normal; KCO, carbon monoxide transfer coefficient, also known as Krogh-Index (DLCO/VA, depicted as percentage of normal), “reference range” refers to local reference values for healthy individuals.

To shed light on the impact of iron homeostasis in PH we performed correlation analysis, which uncovered significant associations between iron homeostasis and hemodynamic parameters such as sPAP (systolic pulmonary arterial pressure), TAPSE (tricuspid annular plane systolic excursion), RVEDD (right ventricular end-diastolic diameter), TPG (transpulmonary pressure gradient) and cardiac index (S3 Table).

**Fig 1:**
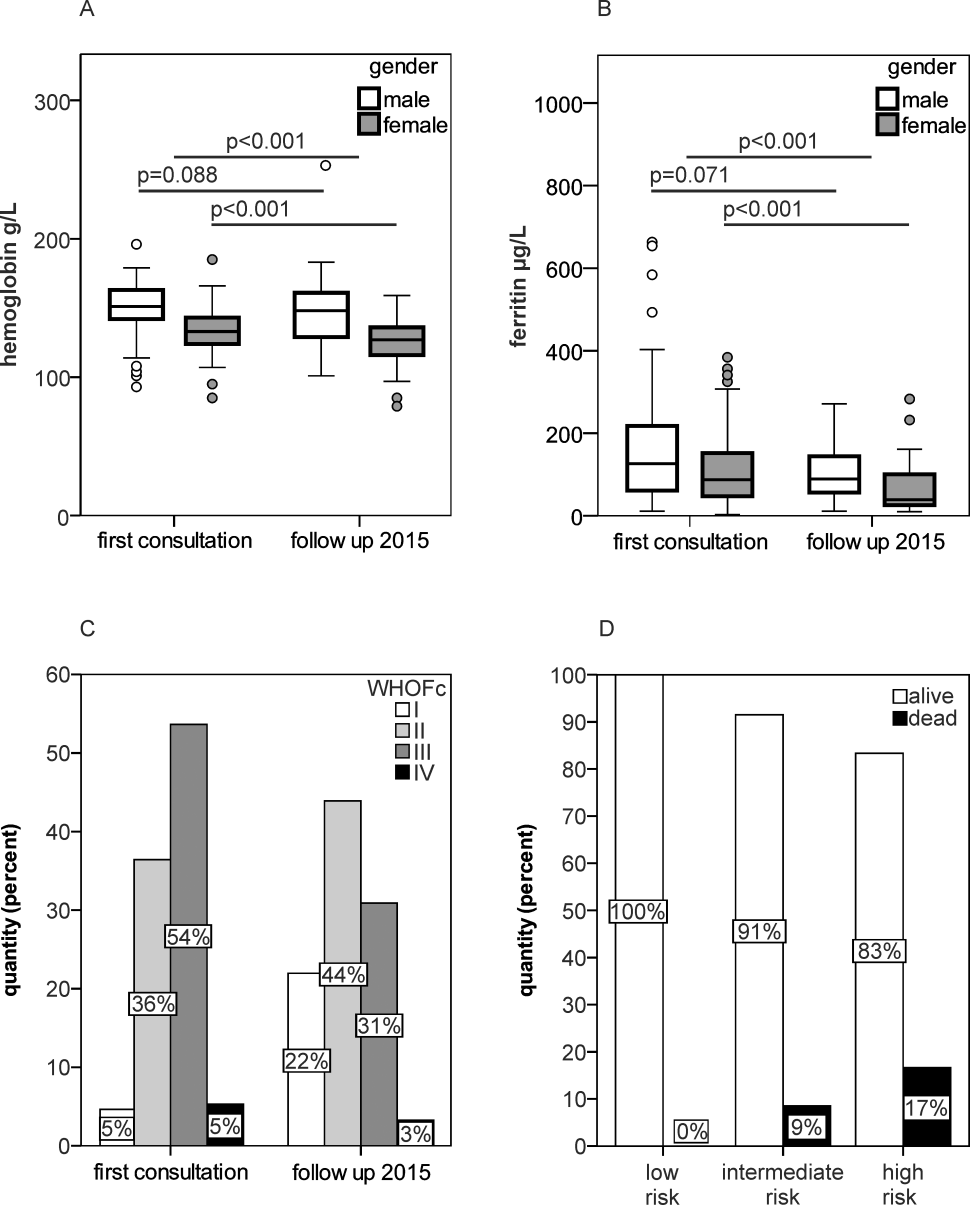
Patients' hematological and clinical parameters at baseline and follow up. (A-B) Time course of hemoglobin (A) and serum ferritin (B) according to gender, (C) WHO functional class (WHOFc) in precapillary PH patients, (D) mortality during the observation period (2006–2016) according to risk assessment at first consultation. N at first consultation = 153 (female = 90/male = 63), N during follow up 2015 = 103 (female = 65/male = 38). Statistical analysis for time-dependent changes was calculated for the 103 patients available at both visits.

Upon re-evaluation of patients, we identified a significant decrease in hemoglobin levels (p<0.001) and serum ferritin levels (p<0.001) over time (S4 Table, Fig 1A and 1B). Hemodynamic parameters such as PVR (pulmonary vascular resistance), TPG and sPAP showed a significant decrease, whereas cardiac index and TAPSE levels remained unchanged from the first consultation to re-evaluation. At follow up, patients demonstrated improved WHOFc as compared to baseline (p<0.001) (Fig 1C). In contrast, oxygen diffusion capacity, as reflected by Krogh-index (DLCO/VA) measurement, slightly decreased over time (p<0.001). Patients`mortality strongly correlated with the performed risk assessment score (Fig 1D). The observed mean five-year mortality rate was 15.7%.

#### Characterization of anemia and iron deficiency in precapillary pulmonary hypertension patients

**Fig 2:**
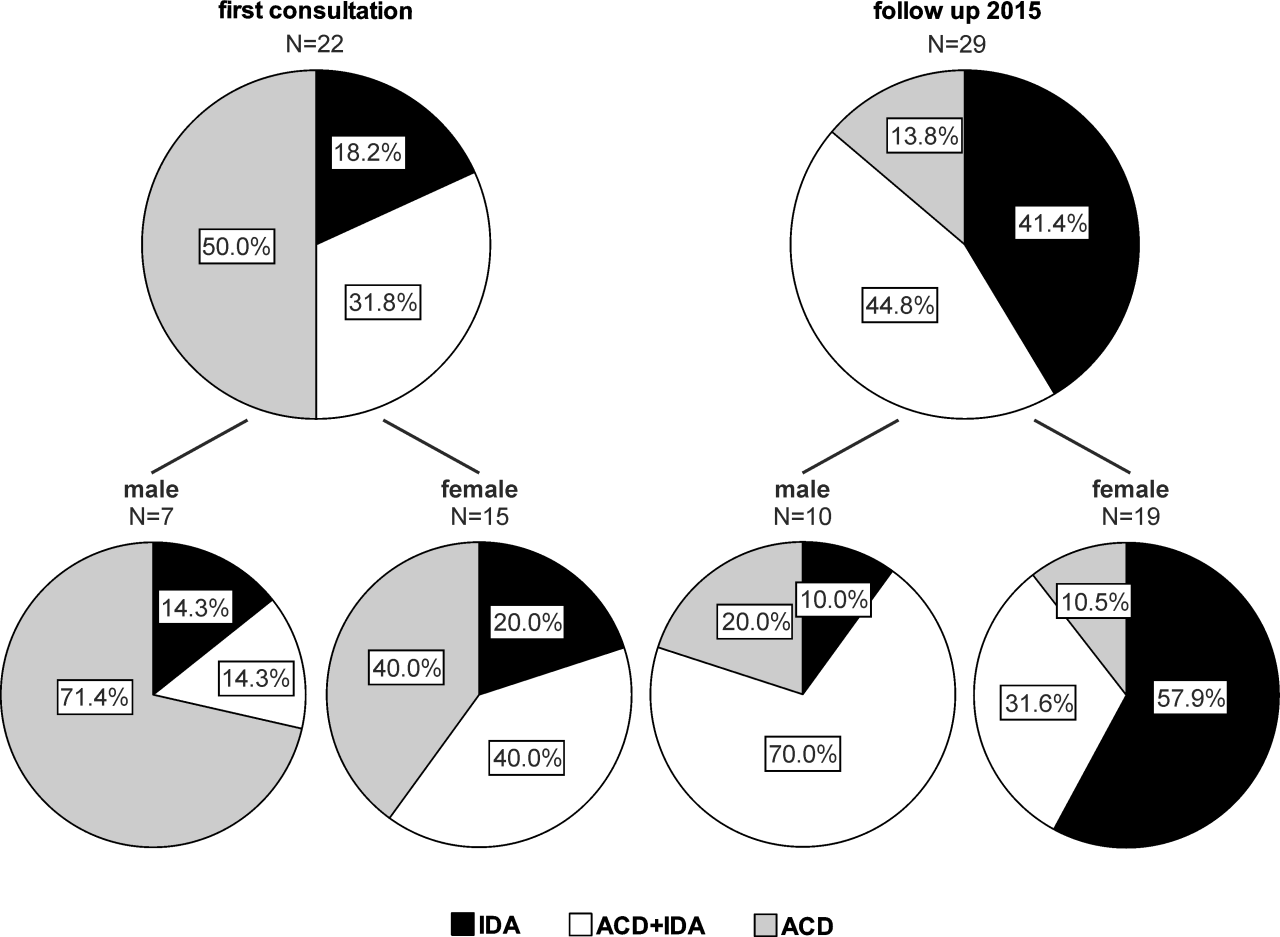
Characterization of anemia in precapillary pulmonary hypertension patients at baseline and follow up. The frequency of iron deficiency anemia (IDA), anemia of chronic disease (ACD) and the combination of both (ACD+IDA) depending on gender at first consultation and follow up in 2015 are shown.

After the evaluation of patients`baseline and follow up characteristics, we further delineated ID and anemia in our patient cohort. Anemia was found in 17.1% of the patients at initial presentation and its frequency significantly rose to 28.2% at the follow up (p<0.001). Anemia was more prevalent in postmenopausal female than in male patients at first presentation (19.2% vs 13.7%) and then increased in both genders in a comparable fashion. Based on previously reported definitions [1], most anemic PH patients were identified as having ACD or ACD+IDA at the initial presentation, whereas IDA and ACD+IDA became more prominent at follow up (Fig 2). Notably, the use of PAH-specific medications such phosphodiesterase type 5 (PDE5) inhibitors, endothelin receptor antagonists (ETRA), guanylate cyclase (GC) stimulators or prostacyclin analogs did not significantly impact on the frequency of anemia or ID in our patient cohort.

Whereas the definition and categorization of anemia are based on rather robust parameters, previous clinical trials used several different definitions of ID [9, 11, 29, 30].

Most of these do not differentiate between true/absolute and functional ID, although they have contrasting underlying pathophysiologies [1]. We assumed that these diverse definitions of ID may display significant discrepancy in their specificity and sensitivity to describe ID. Thus, we studied five different but clinically used ID definitions (ID1-5) to analyze the frequency and clinical impact of ID in our patient cohort. According to the available data and observed mortality a total of 142 at first consultation, 103 in 2015 and 101 patients in 2016 were eligible for further evaluation.

**Fig 3:**
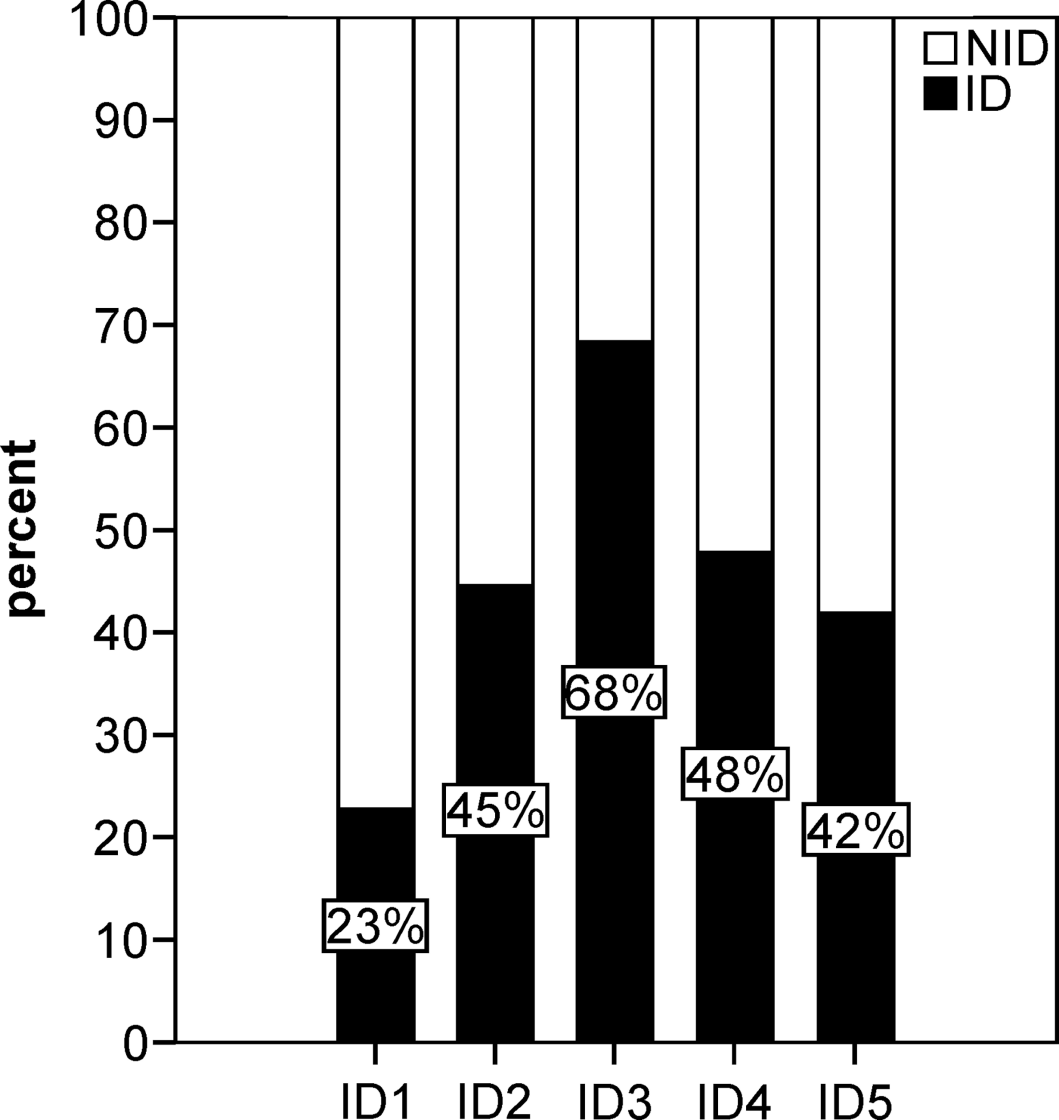
Frequency of iron deficiency in precapillary pulmonary hypertension patients according to differential iron deficiency definitions. Frequency of iron deficiency (ID) according to TSAT and serum ferritin based (ID1-3) and sTFR based ID definitions (ID4-5) at last follow up (N = 101). ID1, serum ferritin <30μg/L and TSAT<16%; ID2, serum ferritin <100μg/L and TSAT<20%; ID3, serum ferritin <100μg/L or serum ferritin 100–299μg/L and TSAT<20%, ID4, sTFR >4.5 for women and >5.0 for men; ID5, sTFRF index >3.2 if CRP <0.5mg/dL or sTFRF index >2 if CRP >0.5mg/dL.

The prevalence of ID highly varied depending on the definitions used (Fig 3). Whereas sTFR and sTFRF index (ID4, ID5) revealed ID in 33% to 48% of patients, the TSAT/ferritin based definitions displayed 11% of patients as being truly iron deficient (ID1). The inclusion of patients with true and functional ID according to ID3 categorized 75% of PH patients as iron deficient. According to these results, we implemented additional ROC analysis to evaluate the sensitivity and specificity of different ID definitions. The sTFRF index is currently considered the most sensitive and specific clinical definition for ID even in the setting of inflammation [32], thus we used ID5 as a reference for ROC analysis (S1 Fig). The sTFR as a single marker (ID4) demonstrated a high association with ID5 (sensitivity of 83.3% and specificity of 79.9%, respectively). The diagnostic accuracy of the definitions using serum ferritin and TSAT (ID1-3) was significantly lower (S1 Fig), whereas ID3 demonstrated the lowest accuracy to correctly diagnose ID as compared to ID5.

#### Impact of iron deficiency on hemodynamic, laboratory and clinical outcome parameters in precapillary PH patients

**Fig 4:**
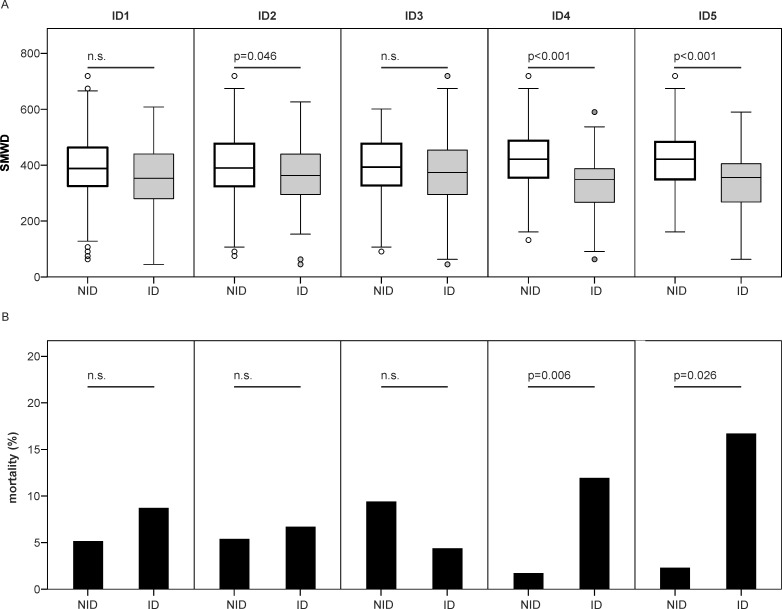
Six minute walking distance and mortality according to differential definitions of iron deficiency. (A) Six minute walking distance (SMWD) at follow up in 2015 according to differential definitions of iron deficiency (ID), (B) overall mortality during observation time according to differential definitions of ID. ID1-3 report serum ferritin and TSAT based ID definitions, whereas ID4 and ID5 present sTFR based ID definitions. ID1, serum ferritin <30μg/L and TSAT<16%; ID2, serum ferritin <100μg/L and TSAT<20%; ID3, serum ferritin <100μg/L or serum ferritin 100–299μg/L and TSAT<20%, ID4, sTFR >4.5 for women and >5.0 for men; ID5, sTFRF index >3.2 if CRP <0.5mg/dL or sTFRF index >2 if CRP >0.5mg/dL, N = 103 (A), N = 153 (B).

Interestingly, ID according to differential ID definitions had a diverse impact on the outcome in PH. Clinical performance parameters, such as SMWD, and hemodynamic parameters, such as the cardiac index and mPAP (mean pulmonary arterial pressure), as well as serum NT-proBNP (N-terminal pro-B-type natriuretic peptide) levels were significantly different when comparing NID PH patients to ID PH patients using ID definitions ID2, ID4 and ID5 (Table 2, S5 and S6 Tables). Moreover, patients with ID according to the sTFRF index or sTFR displayed significantly lower DLCO levels and more frequently needed long-term oxygen treatment. We found a significant correlation of ID with PH mortality risk assessment and observed a higher mortality in ID patients merely for sTFR based ID definitions (ID4, ID5), which was not seen when using ID1-3 (Table 2 and Fig 4). In detail, according to ID4 and ID5 definitions, an overall mortality of 11.9% and 10.4% in ID patients as compared to a mortality of 1.7% and 1.9% in NID patients was observed, respectively.

**Table 2:** Patients' characteristics according to the sTFRF index at follow up.

	NID N = 44			ID N = 30			NID vs ID		NID N = 44			ID N = 30			NID vs ID
	mean	±	SD	mean	±	SD	p-value		mean	±	SD	mean	±	SD	p-value
**observation time** (months)	67.9	±	45.4	73.2	±	33.8	0.339	**right heart catheterization**							
								PAPm (mmHg)	34.9	±	10.8	41	±	10.8	**0.017**
**laboratory blood tests**								RAPm (mmHg)	9.3	±	4.7	12.2	±	4.5	**0.008**
hemoglobin (g/l)	135.2	±	19.7	128.7	±	19.8	0.075	Cardiac index (L/min/m^2^)	2.7	±	0.7	2.3	±	0.7	**0.013**
RDW (%)	14.1	±	1.3	15.9	±	2.2	**<0.001**	PCWP (mmHG)	14.9	±	7	16.4	±	5.4	0.178
MCV (fL)	87.6	±	10.1	85.6	±	6.4	**0.012**	PVR (dynxsxcm-5)	423.0	±	225.6	579.1	±	316.7	0.064
MCH (pg)	33	±	11	28.2	±	3	**0.002**	SvO2 (%)	68.3	±	7.5	66.7	±	10.9	0.461
sTFR (mg/L)	3.3	±	1.1	5.8	±	2.5	**<0.001**	TPG (mmHG)	20.0	±	10.7	24.8	±	10.5	**0.029**
serum iron (μmol/L)	16.8	±	6.9	12.3	±	4.9	**0.014**								
transferrin (mg/dL)	250.9	±	37.9	268.7	±	72.2	**0.036**	**echocardiography**							
transferrin saturation (%)	26.5	±	10.3	16.2	±	6.8	**<0.001**	sPAP (mmHg)	46.9	±	17	56.7	±	25.1	0.124
ferritin (μg/L)	98.3	±	89.7	41.0	±	34.3	**<0.001**	TAPSE (mm)	22.1	±	7.8	22.0	±	6.1	0.966
NT-proBNP (ng/L)	382	±	566	1327	±	1468	**0.001**	RVEDD (mm)	32.3	±	9.9	36.2	±	9.5	0.124
CRP (mg/dL)	0.4	±	0.4	0.7	±	0.5	**0.003**	LVEF (%)	59.6	±	7.6	56	±	10.2	0.07
GFR mL/min/1.73m^2^)	51.0	±	17.4	50.3	±	12.3	0.334	RA area (cm^2^)	16.9	±	9.8	18.3	±	6.4	0.286
uric acid (mg/dL)	6.0	±	1.6	7.0	±	2.3	0.122								
creatinine (mg/dL)	1.0	±	0.3	2.8	±	7.1	**0.015**	**pulmonary function test**							
								DLCO (%)	72.3	±	20.7	58.3	±	23.6	**0.034**
**arterial blood gas analysis**								KCO (%)	83.1	±	25.4	76.1	±	27.2	0.214
pO2 (mmHg)	66.9	±	14.7	65.9	±	11.2	0.362								
pCO2 (mmHg)	37.0	±	8.6	38.1	±	9.1	0.585	**risk assessment PH associated 1-year mortality**	**N (%)**			**N (%)**			**0.001**
AaDO2 (mmHg)	30.7	±	10.8	33	±	12.5	0.346	low risk (<5%)	27 (65.9)			7 (24.1)			
								intermediate risk (5–10%)	12 (29.3)			15 (51.7)			
**SMWD** (m)	422.9	±	112	338.4	±	132.2	**0.018**	high risk (>10%)	2 (4.9)			7 (24.1)			

Data are represented as mean ± 1 standard deviation (SD); N depicts number of valid data for retrospective analysis; abbreviation: sTFRF index, soluble transferrin receptor/log serum ferritin index; NID, non-iron deficient; ID iron deficient (defined as sTFRF index>3.2 if CRP <0.5 mg/dL or sTFRFI >2 if CRP >0.5 mg/dL); RDW, red blood cell distribution width; MCV, mean corpuscular volume; MCH, mean corpuscular hemoglobin; sTFR, soluble transferrin receptor; NTproBNP, N-terminal pro-B-type natriuretic peptide; CRP, C reactive protein; GFR, glomerular filtration rate; pO2, arterial partial pressure of oxygen; pCO2, arterial partial pressure of carbon dioxide; AaDO2, alveolar-arterial oxygen difference; SMWD, six minute walking distance; PAPm, mean pulmonary arterial pressure; RAPm, mean right atrial pressure; PCWP, pulmonary capillary wedge pressure; PVR, pulmonary vascular resistance; SvO2, mixed venous saturation; TPG, transpulmonary pressure gradient (PAPm-PCWP); sPAP, systolic pulmonary arterial pressure; TAPSE, tricuspid annular plane systolic excursion; RVEDD, right ventricular end diastolic diameter; LVEF, left ventricular ejection fraction; DLCO, diffusing capacity for carbon monoxide, depicted as percentage of normal; KCO, carbon monoxide transfer coefficient, also known as Krogh-Index (DLCO/VA, depicted as percentage of normal). Reference values of healthy individuals are depicted in Table 1.

We also evaluated the impact of ID according to the PH group, as the differential biology seen in these subgroups of PH might alter the effect of ID on clinical outcome parameters. Group 3 PH patients demonstrated a significantly shorter survival time than patients of other PH groups (p<0.001). In line with previous analyses, mortality was significantly increased in PH group 1 patients with ID as compared to NID patients when applying ID4 or ID5 definitions (p = 0.037 and p = 0.049, respectively). ID according to ID1-3 definitions did not affect mortality in group 1 PH patients. We did not observe significant differences in mortality due to ID in PH patients of group 2–5, however, the value of these analyses was limited by the low numbers of diseased patients in PH groups 2–5.

#### Impact of inflammatory markers and hepcidin on iron deficiency and clinical outcome parameters in PH patients

**Fig 5:**
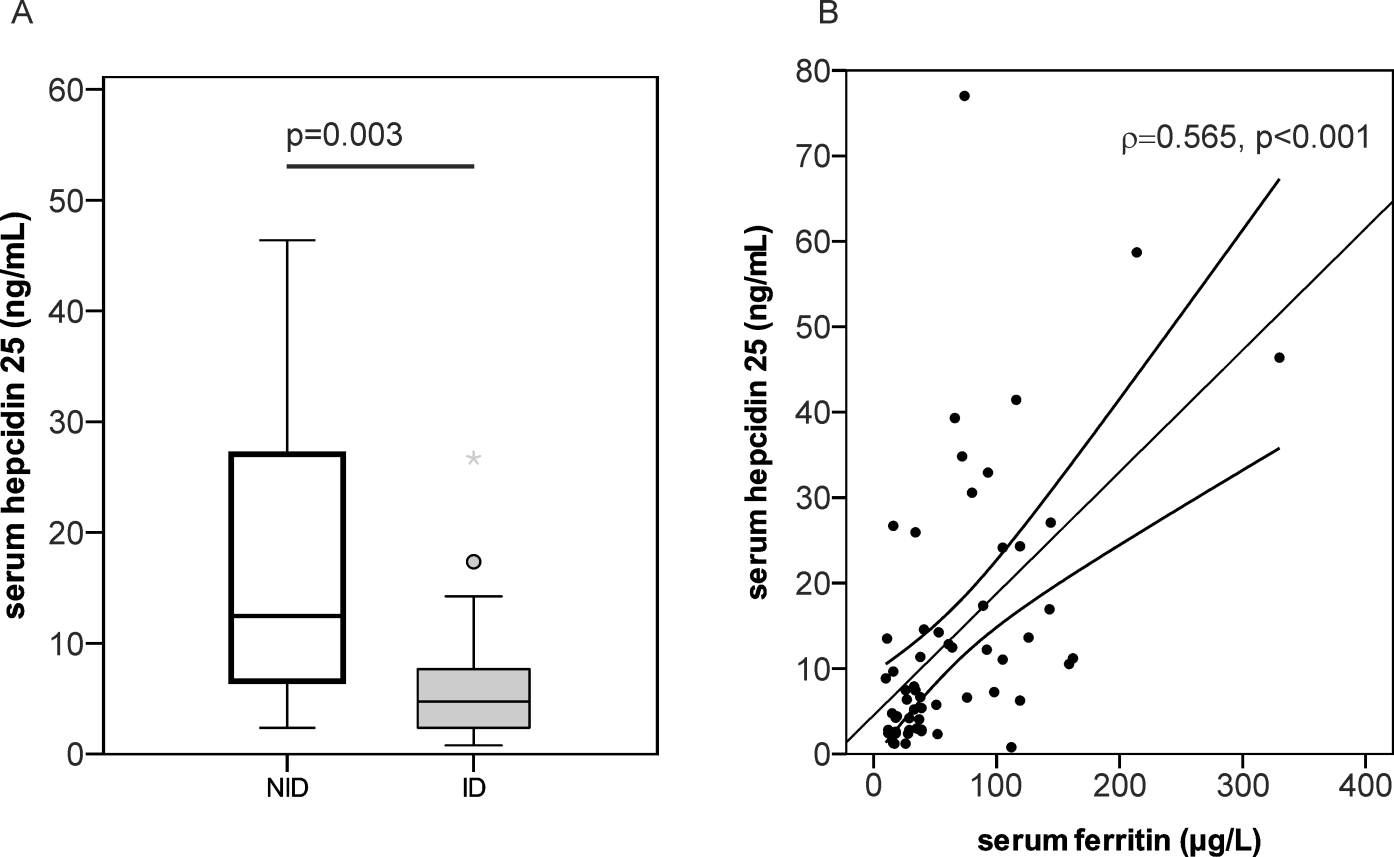
Serum hepcidin in iron deficient (ID) and non-iron deficient (NID) precapillary pulmonary hypertension patients. (A) Serum hepcidin 25 expression in iron deficient (ID) and non-iron deficient (NID) patients. (B) Correlation of serum hepcidin 25 with serum ferritin. ID was defined according to the soluble transferrin receptor/logferritin index (sTFRF index, ID5: sTFRF index >3.2 if CRP <0.5mg/dL or sTFRF index >2 if CRP >0.5mg/dL); N = 45; ρ, Spearman rho correlation coefficient; p, p-value as reported by Spearman rho test.

Signs of inflammation, as reflected by CRP levels above 0.7mg/dL, were detected in 32.9% of patients at first consultation and 29.0% of all patients at follow up, respectively. To gain more insight into mechanisms underlying ID in PH patients and how it affects the clinical course, we performed a subgroup analysis including determination of hepcidin, CRP and IL6 in the serum of 45 PH patients. Serum hepcidin levels were significantly lower in ID than in NID subjects (Fig 5) according to all ID definitions, and serum hepcidin levels correlated with a lower RDW and a higher MCH (S7 Table). Notably, markers of inflammation did not correlate with serum hepcidin levels (Fig 5 and S7 Table), suggesting that serum hepcidin levels were mainly influenced by iron availability, hypoxia and hematopoietic factors [33]. Notably, there was no association between serum hepcidin with clinical outcome parameters such as SMWD or patients`survival (S7 Table).

#### Iron deficiency affects the clinical outcome in precapillary pulmonary hypertension independent of the emergence of anemia

**Fig 6:**
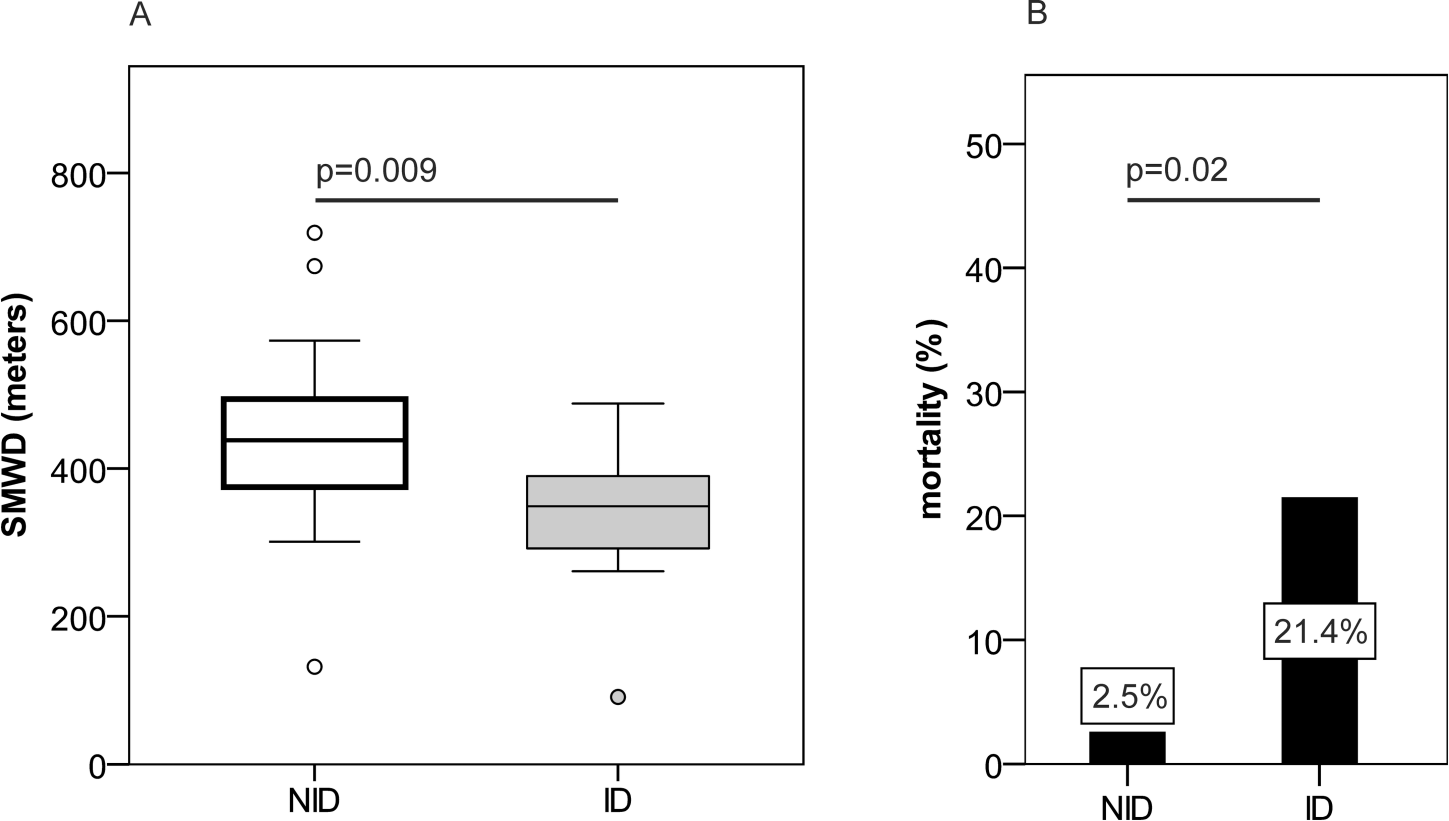
SMWD and mortality of non-anemic precapillary PH patients depending on iron status at follow up in 2015. (A) Six-minute walking distance (SMWD) and (B) observed mortality in non-iron deficient (NID) and iron deficient (ID) patients with precapillary pulmonary hypertension as defined by ID4 at follow up in 2015; N = 54.

Anemia was previously associated with a worse clinical outcome of PH patients [34], thus anemia may be a relevant driver of increased mortality and impaired performance status of PH patients. As this poses a relevant issue for treatment considerations, we evaluated clinical outcome parameters in ID and NID PH subjects, who did not display anemia during the observation period. Notably, clinical performance parameters, such as the SMWD, and patients’ mortality were significantly deteriorated in ID non-anemic precapillary PH patients as compared to non-anemic NID PH individuals, when applying sTFR based ID definitions (Fig 6). The latter was not found when using ID1-3. To conclude, ID affected the clinical outcome of precapillary PH patients also in the absence of anemia and sTFR dependent ID definitions ID4 and ID5 demonstrated to be the best predictors for ID associated clinical impairment.

## Discussion

ID has been linked to PH, however, the causal relationship is still elusive [35]. One major problem in this regard is the varying definition of ID. Specifically, some definitions include patients with true/absolute and functional/inflammation driven ID, which have contrasting underlying pathophysiologies. Therefore, it is impossible to draw any conclusion on the effect of such different alterations of iron homeostasis on the course of PH. We systematically compared five different definitions of ID and evaluated their clinical usefulness in PH.

The prognostic value of several hemodynamic, serological and clinical parameters in our patient cohort is in line with published data [36]. Over time, we observed a significant decline in serum ferritin and hemoglobin levels, suggesting that ID aggravates in the course of PH. Of note, anemia in PH patients was found in up to one-third of subjects, with ACD either alone or in combination (ACD+IDA) being most prevalent. This observation indicates already, that alterations of iron homeostasis in PH subjects are of multifactorial nature and that inflammation driven diversion of iron traffic is a dominant reason for anemia. The presence of both, anemia and/or inflammation, was associated with an adverse outcome of patients, suggesting that multiple pathways affect the course of PH patients [37].

Of note, many more patients presented with alterations of iron homeostasis than with anemia. When applying five different definitions of ID, we found major differences in the relative number of patients being diagnosed as iron deficient ranging from 11% to 75%. However, ID according to several definitions affected clinical outcome and performance status independent of the presence/absence of anemia, which is in line with previous investigations in PAH patients [29], and a study in patients with chronic heart failure [9]. The mechanisms linking ID to impaired exercise capacity even in the absence of anemia are elusive but may be attributed to effects of iron on mitochondrial respiration, cellular metabolism or immune function [22, 38, 39].

A strict definition such as ID1 selects only patients with absolute/true ID, which undoubtedly need iron supplementation. However, this definition may exclude many subjects who require iron, because cut-offs for ID in patients with chronic disease and hypoxia, such as PH patients, may be higher. Oppositely, a too liberal definition of ID, as in ID3, including subjects with true and functional ID, harbors the risk of over-treatment, because we do not know whether patients with PH and true ID versus functional/inflammation driven ID may benefit from iron therapy in a comparable fashion. Based on our study with the presented PH patient cohort a liberal definition of ID by ID3 has a poor association with the course of PH, whereas sTFR based ID definitions (ID4 and ID5) had the most significant association with clinical performance and outcome of our patients.

Given the mentioned limitations of currently used ID definitions in the clinical setting, we implemented the sTFRF index and measured serum hepcidin levels to allow a more robust determination of iron availability in PH subjects. Elevated serum hepcidin levels have been described in PAH patients [29, 31], whereas we found normal to low serum hepcidin levels in the ID PH subgroup. The discrepancy between these results may be due to the fact that serum hepcidin levels are influenced by numerous factors such as ID, hypoxia, inflammation, genetics, erythropoiesis, and medications. For instance, tyrosine kinase inhibitors and diuretics (e.g. spironolactone) reduce serum hepcidin levels [40]. Of note, ID in the setting of inflammation decreases hepcidin expression, because ID impairs hepcidin inducing SMAD pathways [33, 41]. This goes along with our observations that a significant proportion of anemic patients in this cohort suffered from ACD plus IDA, which demonstrate reduced serum hepcidin levels [42].

Recent evaluations of iron homeostasis in PH patients used sTFR to determine ID [29, 31]. An inclusion of ferritin levels into a sTfR/log ferritin ratio, called sTFRF index, provided an even more sensitive and specific method to detect ID and distinguish functional from true ID [43]. The CRP corrected sTFRF index takes inflammatory triggers into account [1, 14, 43], and is useful in patients with comorbidities and chronic inflammatory status, as seen in PH subjects investigated in this study. However, the clinical use of the sTFR and the sTFRF index are currently limited, because sTFR concentrations are not widely performed in clinical routine and cut off levels for the sTFR and sTFRF index depend on the used tests.

## Conclusion

We herein demonstrate that sTFR based definitions of ID are closely linked to the course and outcome of PH subjects. However, our data urge the establishment of an accurate ID definition by means of prospective trials, which should provide more insights into the association of altered iron homeostasis with the pathogenesis and course of PH. Such studies are essential to draw any conclusion on the potential benefits of iron treatment, either as orally or intravenously applied drugs, and will be mandatory to get insights into the effects of iron treatment on the course of PH.

## Supporting information

S1 Table Patients' characteristics at first consultation–PH classification, clinical performance status and mortality risk assessment. (DOCX)

S2 Table Patients' characteristics at first consultation–comorbidities and treatment modalities. (DOCX)

S3 Table Correlations of serum parameters of iron homeostasis with Patients' baseline characteristics. (DOCX)

S4 Table Patients' characteristics time course. (DOCX)

S5 Table Patients' characteristics according to differential serum ferritin and TSAT based definitions of ID at first consultation. (DOCX)

S6 Table Patients' characteristics according to differential definitions of ID at follow up in 2015. (DOCX)

S7 Table Correlations of serum hepcidin with Patients' characteristics at follow up in 2015. (DOCX)

S1 Fig Receiver operating characteristic analysis of differential definitions for iron deficiency. Detection of iron deficiency (ID) using soluble transferrin receptor/logferritin index (sTFRF index) was performed for pulmonary arterial hypertension patient evaluation in 2015. Other definitions for ID were compared to the sTFRF index based ID categorization (N = 103). The following definitions for ID were used: sTFRF index >3.2 if CRP <0.5 mg/dL or sTFRF index >2 if CRP >0.5 mg/dL (reference), ID1, serum ferritin <30μg/L and TSAT<16%; ID2, serum ferritin <100μg/L and TSAT <20%; ID3, serum ferritin <100μg/L or serum ferritin 100–299μg/L and TSAT<20%); ID4, sTFR>4.5 for women and >5.0 for men.
(TIF)
