# Supplementary material for: The crucial impact of iron deficiency definition for the course of precapillary pulmonary hypertension
Source: PLoS One. 2018 Aug 30;13(8):e0203396. doi: 10.1371/journal.pone.0203396 (PMC6117062; doi:10.1371/journal.pone.0203396)
Supplement: S1 Table — (DOCX) [file pone.0203396.s001.docx]

**S1 Table. Patients` characteristics at first consultation – PH classification, clinical performance status and mortality risk assessment.**

| **PH Classification (NICE), N=151** | **N (%)** | | |  | |
| --- | --- | --- | --- | --- | --- |
| Type 1 | 93 (61.6) | | |  | |
| Type 2 | 16 (10.6) | | |  | |
| Type 3 | 5 (3.3) | | |  | |
| Type 4 | 30 (19.9) | | |  | |
| Type 5 | 7 (4.6) | | |  | |
|  |  | |  | |  |
| **WHOFc, N=151** | **N (%)** | |  | |  |
| I (N/%) | 7 (4.6) | |  | |  |
| II (N/%) | 55 (35.9) | |  | |  |
| III (N/%) | 81 (52.9) | |  | |  |
| IV (N/%) | 8 (5.2) | |  | |  |
|  |  |  |  | |  |
| **risk assessment PH Mortality (ERS), N=101** | **N (%)** |  |  | |  |
| low risk (<5% mortality/year) | 42 (41.6) | | | |  |
| intermediate risk (5-10% mortality/year) | 47 (46.5) | | | |  |
| high risk (>10% mortality/year) | 12 (11.9) | | | |  |

N depicts the number of valid data for retrospective analysis; PH, pulmonary hypertension, WHOFc, WHO functional class; Type 1, pulmonary arterial hypertension; Type 2, pulmonary hypertension due to left heart disease; Type 3, pulmonary hypertension due to lung disease and/or hypoxemia; Type 4, chronic thromboembolic pulmonary hypertension (CTEPH); Type 5, pulmonary hypertension due to unclear or multifactorial conditions.
