# Supplementary material for: The crucial impact of iron deficiency definition for the course of precapillary pulmonary hypertension
Source: PLoS One. 2018 Aug 30;13(8):e0203396. doi: 10.1371/journal.pone.0203396 (PMC6117062; doi:10.1371/journal.pone.0203396)
Supplement: S2 Table — (DOCX) [file pone.0203396.s002.docx]

**S2 Table. Patients` characteristics at first consultation – comorbidities and treatment modalities.**

|  | **percentage of all patients (N=153)** |
| --- | --- |
| **reasons for iron deficiency independent of PH at first consultation** | 85.5% |
| liver cirrhosis | 5.3% |
| autoimmune diseases | 18.7% |
| renal insufficiency | 38.2% |
| microhematuria | 15.8% |
| hematological neoplasia or cancer | 22.4% |
| gynecological/genital bleeding | 10.5% |
| clinical signs of gastrointestinal bleeding | 7.8% |
| positive test for occult blood in the stool | 9.2% |
| positive signs of bleeding in gastroscopy | 12.9% |
| positive signs of bleeding in colonoscopy | 13.6% |
|  |  |
| **treatment during observation time (2006-2016)** |  |
| iron supplementation | 11.2% |
| anticoagulation therapy | 70.1% |
| LTOT | 31.0% |
| PAH-specific treatment at first consultation | 8.5% |
| PAH-specific treatment during the observation time | 86.7% |
| mono-therapy | 56.7% |
| combination-therapy | 30.1% |
| PDE5 inhibitor | 45.3% |
| ETRA | 52.3% |
| GC stimulator | 7.5% |
| prostacyclin analoges | 7.5% |

N depicts the number of valid data for retrospective analysis; abbreviations: PAH, pulmonary arterial hypertension; LTOT, long-term oxygen treatment; PAH-specific treatment: endothelin receptor antagonists (ETRA), phosphodiesterase type 5 (PDE5) inhibitors, guanylate cyclase (GC) stimulators, prostacyclin analogues; combination therapy: patients receiving two or more specific PAH drugs.
