# Supplementary material for: The crucial impact of iron deficiency definition for the course of precapillary pulmonary hypertension
Source: PLoS One. 2018 Aug 30;13(8):e0203396. doi: 10.1371/journal.pone.0203396 (PMC6117062; doi:10.1371/journal.pone.0203396)
Supplement: S3 Table — (DOCX) [file pone.0203396.s003.docx]

**S3 Table. Correlations of serum parameters of iron homeostasis with patients` baseline characteristics.**

|  | **serum iron** | **serum transferrin** | **transferrin saturation** | **serum ferritin** |
| --- | --- | --- | --- | --- |
|  | **Spearman RHO`s correlation coefficient** | | | |
| **clinical and laboratory parameters** | | | | |
| age (years) | 0.115 | -0.133 | 0.124 | 0.173 |
| bodyweight (kg) | 0.096 | -.178^*^ | 0.101 | **0.245**** |
| BMI (kg/m^2^) | -0.036 | 0.058 | 0.063 | 0.087 |
| hemoglobin (g/l) | **0.239*** | 0.141 | **0.266**** | **0.189*** |
| RDW (%) | **-0.376**** | 0.083 | **-0.276**** | -0.136 |
| MCV (fL) | **0.277*** | -0.024 | **0.336***** | **0.211**** |
| MCH (pg) | **0.508***** | -0.003 | **0.404***** | **0.227**** |
| NTproBNP (ng/L) | 0.141 | 0.005 | 0.089 | 0.165 |
| CRP (mg/dL) | -0.193 | **-0.304**** | -0.048 | 0.122 |
| GFR mL/min/1.73m^2^) | -0.055 | 0.105 | 0.088 | 0.003 |
| uric acid (mg/dL) | 0.172 | 0.101 | 0.076 | **0.210*** |
| creatinine (mg/dL) | 0.107 | -0.158 | 0.078 | **0.244**** |
| pO2 (mmHg) | 0.065 | -0.012 | 0.043 | -0.010 |
| pCO2 (mmHg) | **-0.231*** | -0.037 | -0.176 | -0.054 |
| AaDO2 (mmHg) | 0.138 | 0.085 | 0.053 | 0.054 |
| DLCO (%) | 0.106 | 0.163 | -0.117 | 0.044 |
| KCO (%) | 0.004 | 0.112 | -0.070 | 0.037 |
| SMWD (m) | -0.148 | -0.124 | -0.014 | 0.040 |
| WHOFc | -0.152 | 0.095 | -0.125 | -0.141 |
|  |  |  |  |  |
| **right heart catheterization** |  |  |  |  |
| PAPm (mmHg) | 0.145 | **0.184*** | 0.017 | 0.029 |
| RAPm (mmHg) | 0.142 | 0.105 | 0.048 | 0.074 |
| Cardiac index (L/min/m^2^) | -0.175 | **-0.215*** | -0.026 | -0.016 |
| PCWP (mmHG) | 0.038 | -0.043 | -0.079 | 0.031 |
| PVR (dynxsxcm-5) | 0.117 | 0.180 | 0.039 | -0.027 |
| SvO2 (%) | -0.119 | -0.061 | -0.075 | -0.113 |
| TPG (mmHG) | 0.145 | **0.212*** | 0.023 | -0.060 |
|  |  |  |  |  |
| **echocardiography** |  |  |  |  |
| sPAP (mmHg) | **0.287*** | 0.091 | **0.218*** | 0.165 |
| TAPSE (mm) | -0.158 | **-0.304*** | -0.120 | -0.046 |
| RVEDD (mm) | **0.362**** | 0.042 | **0.205*** | 0.175 |
| LVEF (%) | -0.213 | 0.112 | -0.165 | -0.144 |

Correlations were calculated with Spearman's Rho test and the correlation coefficient is shown. *p-value <0.05. **p-value<0.01. ***p-value <0.001. A significant correlation coefficient between +/- 0.1 to 0.3 was considered weak. from +/- 0.3 to 0.5 moderate and from +/- 0.5 to 1.0 strong; abbreviations: BMI, body mass index; RDW, red blood cell distribution width; MCV, mean corpuscular volume; MCH, mean corpuscular hemoglobin; NT-proBNP, N-terminal pro-B-type natriuretic peptide; CRP, C reactive protein; GFR, glomerular filtration rate; pO2, arterial partial pressure of oxygen; pCO2, arterial partial pressure of carbon dioxide; AaDO2, alveolar-arterial oxygen difference; DLCO, diffusing capacity for carbon monoxide; KCO, carbon monoxide transfer coefficient, also known as Krogh-Index (DLCO/VA); SMWD, six-minute walking distance; EHOFc, WHO functional class; PAPm; mean pulmonary arterial pressure; RAPm, mean right atrial pressure; PCWP, pulmonary capillary wedge pressure; PVR, pulmonary vascular resistance; SvO2, mixed venous saturation; TPG, transpulmonary pressure gradient (PAPm-PCWP); sPAP, systolic pulmonary arterial pressure; TAPSE, tricuspid annular plane systolic excursion; RVEDD, right ventricular end-diastolic diameter; LVEF, left ventricular ejection fraction.
