# Supplementary material for: The crucial impact of iron deficiency definition for the course of precapillary pulmonary hypertension
Source: PLoS One. 2018 Aug 30;13(8):e0203396. doi: 10.1371/journal.pone.0203396 (PMC6117062; doi:10.1371/journal.pone.0203396)
Supplement: S4 Table — (DOCX) [file pone.0203396.s004.docx]

| **S4 Table. Patients‘ characteristics time course.**   \|  \| **First consultation** \| \| \| **Follow up 2015** \| \| \|  \|  \|  \| **First consultation** \| \| \| **Follow up 2015** \| \| \|  \| \| --- \| --- \| --- \| --- \| --- \| --- \| --- \| --- \| --- \| --- \| --- \| --- \| --- \| --- \| --- \| --- \| --- \| \|  \| **N=103** \| \| \| \| \| \|  \|  \|  \| **N=103** \| \| \| \| \| \|  \| \|  \| **mean** \| **±** \| **SD** \| **mean** \| **±** \| **SD** \| **p-value** \|  \|  \| **mean** \| **±** \| **SD** \| **mean** \| **±** \| **SD** \| **p-value** \| \| **hematological parameters** \|  \|  \|  \|  \|  \|  \|  \|  \| **right heart catheterization** \|  \|  \|  \|  \|  \|  \|  \| \| hemoglobin (g/L) \| 141.5 \| ± \| 20.6 \| 133.0 \| ± \| 23.1 \| **<0.001** \|  \| PAPm (mmHg) \| 41.03 \| ± \| 15.6 \| 39.6 \| ± \| 15.4 \| 0.055 \| \| RDW (%) \| 15.0 \| ± \| 2.1 \| 15.2 \| ± \| 2.3 \| 0.401 \|  \| RAPm (mmHg) \| 11.25 \| ± \| 5.4 \| 11.2 \| ± \| 5.4 \| 0.940 \| \| MCV (fL) \| 88.9 \| ± \| 6.1 \| 87.9 \| ± \| 8.4 \| **0.001** \|  \| Cardiac index (L/min/m^2^) \| 2.3 \| ± \| 0.5 \| 2.4 \| ± \| 0.7 \| 0.142 \| \| MCH (pg) \| 29.8 \| ± \| 2.5 \| 30.2 \| ± \| 6.6 \| 0.610 \|  \| PCWP (mmHG) \| 16.1 \| ± \| 7.1 \| 15.8 \| ± \| 6.3 \| 0.636 \| \| serum iron (µmol/L) \| 16.8 \| ± \| 8.0 \| 14.3 \| ± \| 7.0 \| **0.017** \|  \| PVR (dynxsxcm-5) \| 589.3 \| ± \| 428.8 \| 511.2 \| ± \| 310.1 \| **0.019** \| \| transferrin (mg/dL) \| 272.1 \| ± \| 55.8 \| 260.5 \| ± \| 56.7 \| **0.006** \|  \| SvO2 (%) \| 65.3 \| ± \| 8.4 \| 65.4 \| ± \| 9.3 \| 0.743 \| \| transferrin saturation (%) \| 24.3 \| ± \| 13.7 \| 22.7 \| ± \| 12.0 \| 0.204 \|  \| TPG (mmHG) \| 25.0 \| ± \| 15.3 \| 23.2 \| ± \| 14.7 \| **0.012** \| \| ferritin (µg/L) \| 130.4 \| ± \| 121.5 \| 106.8 \| ± \| 225.4 \| **<0.001** \|  \|  \|  \|  \|  \|  \|  \|  \|  \| \| NTproBNP (ng/L) \| 1624.8 \| ± \| 2060.5 \| 1419.1 \| ± \| 2977.6 \| 0.100 \|  \| **echocardiography** \|  \|  \|  \|  \|  \|  \|  \| \| CRP (mg/dL) \| 0.8 \| ± \| 1.6 \| 0.7 \| ± \| 1.1 \| 0.313 \|  \| sPAP (mmHg) \| 59.2 \| ± \| 19.4 \| 53.3 \| ± \| 20.2 \| **0.002** \| \| GFR mL/min/1.73m^2^) \| 54.6 \| ± \| 10.2 \| 58.6 \| ± \| 22.8 \| **<0.001** \|  \| TAPSE (mm) \| 19.3 \| ± \| 6.0 \| 21.7 \| ± \| 6.6 \| 0.774 \| \| uric acid (mg/dL) \| 7.1 \| ± \| 2.2 \| 6.9 \| ± \| 2.0 \| 0.261 \|  \| RVEDD (mm) \| 37.0 \| ± \| 8.2 \| 34.6 \| ± \| 9.2 \| 0.108 \| \|  \|  \|  \|  \|  \|  \|  \|  \|  \| LVEF (%) \| 54.9 \| ± \| 11.0 \| 58.5 \| ± \| 9.2 \| 0.070 \| \| **arterial blood gas analysis** \|  \|  \|  \|  \|  \|  \|  \|  \| **pulmonary function tests & SMWD** \|  \|  \|  \|  \|  \|  \|  \| \| pO2 (mmHg) \| 67.8 \| ± \| 13.3 \| 65.3 \| ± \| 14.3 \| 0.223 \|  \| DLCO (%) \| 71.4 \| ± \| 24.9 \| 64.6 \| ± \| 23.6 \| 0.122 \| \| pCO2 (mmHg) \| 36.6 \| ± \| 6.2 \| 37.0 \| ± \| 8.1 \| 0.231 \|  \| KCO (%) \| 85.5 \| ± \| 29.7 \| 80.0 \| ± \| 27.8 \| **<0.001** \| \| AaDO2 (mmHg) \| 32.3 \| ± \| 12.6 \| 31.4 \| ± \| 13.2 \| 0.647 \|  \| SMWD (m) \| 381.1 \| ± \| 129.6 \| 386.5 \| ± \| 116.9 \| 0.949 \|   Data are represented as mean ± 1 standard deviation (SD); N depicts the number of valid data for retrospective analysis; abbreviations: RDW, red blood cell distribution width; MCV, mean corpuscular volume; MCH, mean corpuscular hemoglobin; NTproBNP, N-terminal pro-B-type natriuretic peptide; CRP, C reactive protein; GFR, glomerular filtration rate; pO2, arterial partial pressure of oxygen; pCO2, arterial partial pressure of carbon dioxide; AaDO2, alveolar-arterial oxygen difference; PAPm, mean pulmonary arterial pressure; RAPm, mean right atrial pressure; PCWP, pulmonary capillary wedge pressure; PVR, pulmonary vascular resistance; SvO2, mixed venous saturation; TPG, transpulmonary pressure gradient (PAPm-PCWP); sPAP, systolic pulmonary arterial pressure; TAPSE, **tricuspid** annular plane systolic excursion; RVEDD, right ventricular end diastolic diameter; LVEF, left ventricular ejection fraction; DLCO, diffusing capacity for carbon monoxide, depicted as percentage of normal; KCO, carbon monoxide transfer coefficient, also known as Krogh-Index (DLCO/VA, depicted as percentage of normal); SMWD, six minute walking distance. |
| --- | --- | --- | --- | --- | --- | --- | --- | --- | --- | --- | --- | --- | --- | --- | --- | --- | --- | --- | --- | --- | --- | --- | --- | --- | --- | --- | --- | --- | --- | --- | --- | --- | --- | --- | --- | --- | --- | --- | --- | --- | --- | --- | --- | --- | --- | --- | --- | --- | --- | --- | --- | --- | --- | --- | --- | --- | --- | --- | --- | --- | --- | --- | --- | --- | --- | --- | --- | --- | --- | --- | --- | --- | --- | --- | --- | --- | --- | --- | --- | --- | --- | --- | --- | --- | --- | --- | --- | --- | --- | --- | --- | --- | --- | --- | --- | --- | --- | --- | --- | --- | --- | --- | --- | --- | --- | --- | --- | --- | --- | --- | --- | --- | --- | --- | --- | --- | --- | --- | --- | --- | --- | --- | --- | --- | --- | --- | --- | --- | --- | --- | --- | --- | --- | --- | --- | --- | --- | --- | --- | --- | --- | --- | --- | --- | --- | --- | --- | --- | --- | --- | --- | --- | --- | --- | --- | --- | --- | --- | --- | --- | --- | --- | --- | --- | --- | --- | --- | --- | --- | --- | --- | --- | --- | --- | --- | --- | --- | --- | --- | --- | --- | --- | --- | --- | --- | --- | --- | --- | --- | --- | --- | --- | --- | --- | --- | --- | --- | --- | --- | --- | --- | --- | --- | --- | --- | --- | --- | --- | --- | --- | --- | --- | --- | --- | --- | --- | --- | --- | --- | --- | --- | --- | --- | --- | --- | --- | --- | --- | --- | --- | --- | --- | --- | --- | --- | --- | --- | --- | --- | --- | --- | --- | --- | --- | --- | --- | --- | --- | --- | --- | --- | --- | --- | --- | --- | --- | --- | --- | --- | --- | --- | --- | --- | --- | --- | --- | --- | --- | --- | --- | --- | --- | --- | --- | --- | --- | --- | --- | --- | --- | --- | --- | --- | --- | --- | --- | --- | --- | --- | --- | --- | --- | --- | --- | --- | --- | --- | --- | --- | --- | --- | --- | --- | --- | --- | --- | --- | --- | --- | --- | --- | --- | --- | --- | --- | --- | --- | --- | --- | --- | --- | --- | --- | --- | --- | --- | --- | --- | --- | --- | --- | --- | --- | --- | --- | --- | --- | --- | --- | --- | --- | --- | --- | --- | --- | --- | --- | --- | --- | --- | --- | --- | --- | --- | --- | --- | --- |
