# Supplementary material for: The crucial impact of iron deficiency definition for the course of precapillary pulmonary hypertension
Source: PLoS One. 2018 Aug 30;13(8):e0203396. doi: 10.1371/journal.pone.0203396 (PMC6117062; doi:10.1371/journal.pone.0203396)
Supplement: S5 Table — (DOCX) [file pone.0203396.s005.docx]

**S5a Table. Patients‘ characteristics according to differential serum ferritin and TSAT based definitions of ID at first consultation.**

|  | **ID1** | | | | | | **ID2** | | | | | | **ID3** | | | | | | **NID vs ID**  **p-value** | | |
| --- | --- | --- | --- | --- | --- | --- | --- | --- | --- | --- | --- | --- | --- | --- | --- | --- | --- | --- | --- | --- | --- |
|  | **NID**  **N=127** | | | **ID**  **N=15** | | | **NID**  **N=104** | | | **ID**  **N=38** | | | **NID**  **N=65** | | | **ID**  **N=77** | | | **ID1** | **ID2** | **ID3** |
| **Gender** |  | | |  | | |  | | |  | | |  | | |  | | |  |  |  |
| female (N (%)) | 71 (55.9) | | | 9 (60.0) | | | 52 (50.0) | | | 28 (73.7) | | | 30 (46.2) | | | 50 (64.9) | | | 0.762 | **0.012** | **0.025** |
| male (N (%)) | 56 (44.1) | | | 6 (40.0) | | | 52 (50.0) | | | 10 (26.3) | | | 35 (53.8) | | | 27 (35.1) | | |  |  |  |
|  |  |  |  |  |  |  |  |  |  |  |  |  |  |  |  |  |  |  |  |  |  |
|  | **mean** | **±** | **SD** | **mean** | **±** | **SD** | **mean** | **±** | **SD** | **mean** | **±** | **SD** | **mean** | **±** | **SD** | **mean** | **±** | **SD** |  |  |  |
| **clinical parameters** |  |  |  |  |  |  |  |  |  |  |  |  |  |  |  |  |  |  |  |  |  |
| age (years) | 68.7 | ± | 12.7 | 57.9 | ± | 15.2 | 69.1 | ± | 13.3 | 63.4 | ± | 12.9 | 69.5 | ± | 13.8 | 65.9 | ± | 13.0 | **0.009** | **0.018** | 0.093 |
| bodyweight kg) | 76.5 | ± | 17.6 | 68.6 | ± | 16.8 | 76.2 | ± | 15.9 | 74 | ± | 21.3 | 78.3 | ± | 15.3 | 73.5 | ± | 19.1 | **0.041** | 0.178 | 0.083 |
| BMI (kg/m^2^) | 26.9 | ± | 5.7 | 26.1 | ± | 6.6 | 26.7 | ± | 5.5 | 26.9 | ± | 6.5 | 27.1 | ± | 5.5 | 26.5 | ± | 5.9 | 0.616 | 0.835 | 0.604 |
|  |  |  |  |  |  |  |  |  |  |  |  |  |  |  |  |  |  |  |  |  |  |
| **laboratory blood tests** |  |  |  |  |  |  |  |  |  |  |  |  |  |  |  |  |  |  |  |  |  |
| hemoglobin (g/L) | 140.5 | ± | 20 | 137 | ± | 33.2 | 142.1 | ± | 21.1 | 135.2 | ± | 23.2 | 144.4 | ± | 22.8 | 136.9 | ± | 20.8 | 0.565 | 0.108 | **0.009** |
| RDW (%) | 14.7 | ± | 1.9 | 17.7 | ± | 2.3 | 14.7 | ± | 2.0 | 15.7 | ± | 2.4 | 14.6 | ± | 2.1 | 15.3 | ± | 2.1 | **<0.001** | **0.035** | **0.019** |
| MCV (fL) | 89.7 | ± | 5.4 | 83.7 | ± | 9.4 | 90.1 | ± | 5.5 | 86.2 | ± | 7.0 | 90.3 | ± | 5.9 | 88.0 | ± | 6.2 | **0.001** | **0.001** | **0.047** |
| MCH (pg) | 30.0 | ± | 2.0 | 26.8 | ± | 3.5 | 30.2 | ± | 1.9 | 28.4 | ± | 3.0 | 30.3 | ± | 2.1 | 29.2 | ± | 2.5 | **<0.001** | **<0.001** | 0.008 |
| serum iron (µmol/L) | 18.0 | ± | 7.7 | 8.1 | ± | 2.6 | 19.7 | ± | 7.7 | 10.3 | ± | 3.7 | 21.3 | ± | 8.3 | 13.0 | ± | 5.3 | **<0.001** | **<0.001** | **<0.001** |
| transferrin (mg/dL) | 257.1 | ± | 44.6 | 342.7 | ± | 65.0 | 255.5 | ± | 44.1 | 295.5 | ± | 67.0 | 247.5 | ± | 45.8 | 281.8 | ± | 56.7 | **<0.001** | **0.001** | **0.001** |
| transferrin saturation (%) | 27.5 | ± | 13.8 | 9.1 | ± | 3.6 | 30.3 | ± | 13.6 | 12.4 | ± | 4.5 | 34.4 | ± | 14.4 | 18.1 | ± | 8.9 | **<0.001** | **<0.001** | **<0.001** |
| ferritin (µg/L) | 159.9 | ± | 142.9 | 18.1 | ± | 7.2 | 182.1 | ± | 148.7 | 43.1 | ± | 26.7 | 240.4 | ± | 157.4 | 64.2 | ± | 46.2 | **<0.001** | **<0.001** | **<0.001** |
| NTproBNP (ng/L) | 2017 | ± | 4596 | 1753 | ± | 2215 | 2250 | ± | 5039 | 1366 | ± | 2076 | 1776 | ± | 1915 | 2146 | ± | 5573 | 0.923 | **0.020** | 0.198 |
| CRP (mg/dL) | 0.9 | ± | 1.6 | 0.4 | ± | 0.3 | 0.9 | ± | 1.7 | 0.7 | ± | 0.7 | 1.0 | ± | 2.0 | 0.7 | ± | 0.9 | 0.162 | 0.877 | 0.91 |
| GFR mL/min/1.73m^2^) | 58.8 | ± | 17.9 | 56.6 | ± | 10.6 | 59.6 | ± | 19.1 | 55.6 | ± | 10.1 | 61.6 | ± | 19.9 | 55.9 | ± | 14.3 | 0.957 | 0.489 | 0.146 |
| uric acid (mg/dL) | 6.9 | ± | 2.1 | 7.1 | ± | 2.6 | 7.1 | ± | 2.1 | 6.7 | ± | 2.3 | 7.3 | ± | 1.8 | 6.6 | ± | 2.4 | 0.784 | 0.22 | **0.033** |
| creatinine (mg/dL) | 1.2 | ± | 0.9 | 1 | ± | 0.4 | 1.2 | ± | 0.9 | 1 | ± | 0.4 | 1.1 | ± | 0.4 | 1.2 | ± | 1.1 | 0.258 | 0.054 | 0.286 |

Data are represented as mean ± 1 standard deviation (SD); N depicts number of valid data for retrospective analysis; ID1, serum ferritin <30µg/L and TSAT <16%; ID2, serum ferritin <100µg/L and TSAT <20%; ID3, serum ferritin <100 µg/L or serum ferritin 100-299 µg/L and TSAT <20 %; abbreviations: BMI, body mass index; RDW, red blood cell distribution width; MCV, mean corpuscular volume; MCH, mean corpuscular hemoglobin; NTproBNP, N-terminal pro-B-type natriuretic peptide; CRP, C reactive protein; GFR, glomerular filtration rate.

**S5b Table. Patients‘ characteristics according to differential serum ferritin and TSAT based definitions of ID at first consultation.**

|  | **ID1** | | | | | | **ID2** | | | | | | **ID3** | | | | | | **NID vs ID**  **p-value** | | | | |
| --- | --- | --- | --- | --- | --- | --- | --- | --- | --- | --- | --- | --- | --- | --- | --- | --- | --- | --- | --- | --- | --- | --- | --- |
|  | **NID**  **N=127** | | | **ID**  **N=15** | | | **NID**  **N=104** | | | **ID**  **N=38** | | | **NID**  **N=65** | | | **ID**  **N=77** | | | **ID1** | | **ID2** | **ID3** | |
| **arterial blood gas analysis** |  |  |  |  |  |  |  |  |  |  |  |  |  |  |  |  |  |  |  | |  |  | |
| pO2 (mmHg) | 69.6 | ± | 13.8 | 63.1 | ± | 12.9 | 69.2 | ± | 14.1 | 68.1 | ± | 13.2 | 68.7 | ± | 13.5 | 69.0 | ± | 14.1 | 0.109 | | 0.698 | 0.909 | |
| pCO2 (mmHg) | 35.9 | ± | 6.1 | 36.5 | ± | 7.9 | 35.6 | ± | 6.1 | 36.8 | ± | 6.9 | 34.9 | ± | 6.4 | 36.8 | ± | 6.2 | 0.719 | | 0.367 | 0.104 | |
| AaDO2 (mmHg) | 30.8 | ± | 12.3 | 37.5 | ± | 16.6 | 31.8 | ± | 11.2 | 30.5 | ± | 16.3 | 33.2 | ± | 10.9 | 30.0 | ± | 14.1 | 0.134 | | 0.715 | 0.216 | |
|  |  |  |  |  |  |  |  |  |  |  |  |  |  |  |  |  |  |  |  | |  |  | |
| **right heart catheterization** | | | | | | | | | | | | | | | | | | | | | | | |
| PAPm (mmHg) | 40.1 | ± | 14.2 | 46.4 | ± | 26.3 | 40.4 | ± | 14.7 | 42.1 | ± | 19.0 | 40.8 | ± | 14.7 | 40.9 | ± | 17.1 | 0.187 | 0.607 | | | 0.815 |
| RAPm (mmHg) | 11.0 | ± | 5.2 | 11.2 | ± | 5.0 | 11.7 | ± | 5.3 | 9.5 | ± | 4.6 | 10.9 | ± | 4.7 | 11.1 | ± | 5.6 | 0.85 | **0.033** | | | 0.892 |
| Cardiac index (L/min/m^2^) | 2.4 | ± | 0.6 | 2.3 | ± | 0.5 | 2.4 | ± | 0.6 | 2.3 | ± | 0.4 | 2.4 | ± | 0.7 | 2.4 | ± | 0.5 | 0.529 | 0.572 | | | 0.667 |
| PCWP (mmHG) | 15.2 | ± | 7.1 | 16.4 | ± | 7.1 | 15.4 | ± | 7.3 | 15 | ± | 6.3 | 14.7 | ± | 6.2 | 15.9 | ± | 7.7 | 0.68 | 0.832 | | | 0.909 |
| PVR (dynxsxcm-5) | 559.9 | ± | 424.4 | 603.7 | ± | 270 | 549.4 | ± | 426.4 | 606.7 | ± | 373.6 | 563.4 | ± | 453.7 | 563.5 | ± | 370.8 | 0.275 | 0.2 | | | 0.669 |
| SvO2 (%) | 65.6 | ± | 8.1 | 63.8 | ± | 7.0 | 64.7 | ± | 8.4 | 66.9 | ± | 6.5 | 65.1 | ± | 8.6 | 65.6 | ± | 7.4 | 0.443 | 0.197 | | | 0.773 |
| TPG (mmHG) | 24.0 | ± | 14.4 | 31.6 | ± | 24.0 | 23.7 | ± | 14.7 | 27.8 | ± | 18.1 | 24.4 | ± | 14.3 | 25.2 | ± | 16.9 | 0.235 | 0.201 | | | 0.931 |
|  | | | | | | | | | | | | | | | | | | | | | | | |
| **echocardiography** | | | | | | | | | | | | | | | | | | | | | | | |
| sPAP (mmHg) | 60.9 | ± | 20.8 | 55.7 | ± | 17.5 | 62.3 | ± | 20.8 | 55.1 | ± | 19.2 | 63.3 | ± | 20.7 | 57.8 | ± | 20.2 | 0.518 | 0.075 | | | 0.105 |
| TAPSE (mm) | 21.0 | ± | 5.8 | 16.4 | ± | 4.9 | 20.7 | ± | 6.3 | 20.6 | ± | 4.1 | 20.1 | ± | 5.4 | 21.2 | ± | 6.1 | 0.057 | 0.737 | | | 0.299 |
| RVEDD (mm) | 37.6 | ± | 8.3 | 37.4 | ± | 8.5 | 38.9 | ± | 8.6 | 34.4 | ± | 6.8 | 38.3 | ± | 7.6 | 37 | ± | 8.9 | 0.968 | **0.009** | | | 0.166 |
| LVEF (%) | 54.7 | ± | 11.2 | 56.7 | ± | 10.3 | 54.2 | ± | 12.0 | 56.6 | ± | 8.5 | 52.9 | ± | 11.8 | 56.6 | ± | 10.2 | 0.62 | 0.399 | | | 0.127 |
|  |  |  |  |  |  |  |  |  |  |  |  |  |  |  |  |  |  |  |  |  | | |  |
| **pulmonary function tests** | | | | | | | | | | | | | | | | | | | | | | | |
| *DLCO (%)* | 66.8 | ± | 25.2 | 70.9 | ± | 28.4 | 67.2 | ± | 24.4 | 67.1 | ± | 28.1 | 66.0 | ± | 22.9 | 67.9 | ± | 26.9 | 0.738 | 0.989 | | | 0.777 |
| *KCO (%)* | 82.1 | ± | 28.4 | 89.7 | ± | 37.0 | 82.5 | ± | 28.9 | 83.5 | ± | 30.7 | 79.1 | ± | 29.9 | 85.6 | ± | 28.8 | 0.637 | 0.949 | | | 0.340 |

Data are represented as mean ± 1 standard deviation (SD); N depicts number of valid data for retrospective analysis; ID1, serum ferritin <30µg/L and TSAT <16%; ID2, serum ferritin <100µg/L and TSAT <20%; ID3, serum ferritin <100 µg/L or serum ferritin 100-299 µg/L and TSAT <20 %; abbreviations: pO2, arterial partial pressure of oxygen; pCO2, arterial partial pressure of carbon dioxide; AaDO2, alveolar-arterial oxygen difference; PAPm, mean pulmonary arterial pressure; RAPm, mean right atrial pressure; PCWP, pulmonary capillary wedge pressure; PVR, pulmonary vascular resistance; SvO2, mixed venous saturation; TPG, transpulmonary pressure gradient (PAPm-PCWP); sPAP, systolic pulmonary arterial pressure; TAPSE, **tricuspid** annular plane systolic excursion; RVEDD, right ventricular end diastolic diameter; LVEF, left ventricular ejection fraction; DLCO, diffusing capacity for carbon monoxide, depicted as percentage of normal; KCO, carbon monoxide transfer coefficient, also known as Krogh-Index (DLCO/VA, depicted as percentage of normal).
