# Supplementary material for: The crucial impact of iron deficiency definition for the course of precapillary pulmonary hypertension
Source: PLoS One. 2018 Aug 30;13(8):e0203396. doi: 10.1371/journal.pone.0203396 (PMC6117062; doi:10.1371/journal.pone.0203396)
Supplement: S6 Table — (DOCX) [file pone.0203396.s006.docx]

**S6 Table. Patients‘ characteristics according to differential definitions of ID at follow up in 2015.**

|  | **ID1** | | | | | | **ID2** | | | | | | **ID3** | | | | | | **p-values (NID vs ID)** | | |
| --- | --- | --- | --- | --- | --- | --- | --- | --- | --- | --- | --- | --- | --- | --- | --- | --- | --- | --- | --- | --- | --- |
|  | **NID (N=82)** | | | **ID (N=21)** | | | **NID (N=56)** | | | **ID (N=47)** | | | **NID (N=21)** | | | **ID (N=82)** | | | **ID1** | **ID2** | **ID3** |
|  | mean | ± | SD | mean | ± | SD | mean | ± | SD | mean | ± | SD | mean | ± | SD | mean | ± | SD |  |  |  |
| **laboratory blood tests** |  |  |  |  |  |  |  |  |  |  |  |  |  |  |  |  |  |  |  |  |  |
| hemoglobin (g/L) | 136.3 | ± | 24.1 | 120.2 | ± | 13.1 | 138.9 | ± | 20.3 | 126.0 | ± | 24.6 | 139.1 | ± | 18.5 | 131.4 | ± | 24.0 | **0.004** | **<0.001** | **0.021** |
| RDW (%) | 15.0 | ± | 2.3 | 16.1 | ± | 2.2 | 14.5 | ± | 1.7 | 16.1 | ± | 2.6 | 14.6 | ± | 2.1 | 15.4 | ± | 2.3 | **0.02** | **0.001** | **0.037** |
| MCV (fL) | 87.5 | ± | 8.9 | 84.5 | ± | 7.3 | 88.0 | ± | 9.5 | 85.6 | ± | 7.4 | 89.9 | ± | 5.7 | 86.2 | ± | 9.1 | **0.046** | **0.015** | 0.07 |
| MCH (pg) | 31.3 | ± | 8.8 | 27.6 | ± | 3.3 | 32.6 | ± | 10.2 | 28.1 | ± | 3.5 | 30.6 | ± | 0.9 | 30.2 | ± | 8.4 | **0.005** | **<0.001** | 0.117 |
| sTfR (mg/L) | 4.0 | ± | 2.1 | 5.3 | ± | 2.2 | 3.5 | ± | 1.1 | 5.3 | ± | 2.8 | 3.3 | ± | 0.9 | 4.5 | ± | 2.4 | **0.005** | **0.002** | **0.037** |
| serum iron (µmol/L) | 14.8 | ± | 7.5 | 12.6 | ± | 4.9 | 17.0 | ± | 7.8 | 11.1 | ± | 4.2 | 20.0 | ± | 8.0 | 13.3 | ± | 6.4 | 0.419 | **<0.001** | **0.004** |
| transferrin (mg/dL) | 249.5 | ± | 41.4 | 301 | ± | 85.3 | 242.7 | ± | 37.5 | 280.5 | ± | 68.3 | 250.3 | ± | 34.3 | 262.9 | ± | 60.8 | **<0.001** | **<0.001** | 0.307 |
| transferrin saturation (%) | 24.0 | ± | 11.1 | 11.0 | ± | 3.6 | 27.6 | ± | 9.7 | 13.9 | ± | 8.2 | 31.2 | ± | 11.7 | 18.9 | ± | 9.9 | **<0.001** | **<0.001** | **<0.001** |
| ferritin (µg/l) | 127.1 | ± | 274.2 | 18.8 | ± | 5.5 | 162.0 | ± | 328.4 | 38.5 | ± | 28.2 | 300.8 | ± | 515.0 | 55.9 | ± | 41.2 | **<0.001** | **<0.001** | **<0.001** |
| NT-proBNP (ng/L) | 1487 | ± | 3274 | 1221 | ± | 1477 | 1006 | ± | 2027 | 1931 | ± | 3785 | 1092 | ± | 1362 | 1499 | ± | 3251 | 0.406 | **0.015** | 0.997 |
| CRP (mg/dL) | 0.8 | ± | 1.2 | 0.5 | ± | 0.5 | 0.6 | ± | 1.3 | 0.9 | ± | 0.9 | 0.5 | ± | 0.5 | 0.8 | ± | 1.2 | 0.446 | 0.057 | 0.262 |
| GFR mL/min/1.73m^2^) | 49 | ± | 15.7 | 51.8 | ± | 12.1 | 52.6 | ± | 13.8 | 46.8 | ± | 15.4 | 49.8 | ± | 19.1 | 49.8 | ± | 14.0 | 0.701 | 0.087 | 0.866 |
| uric acid (mg/dL) | 7.1 | ± | 1.8 | 6.6 | ± | 2.7 | 6.8 | ± | 1.5 | 7.1 | ± | 2.6 | 6.9 | ± | 1.2 | 6.9 | ± | 2.2 | 0.57 | 0.593 | 0.948 |
| creatinine (mg/dL) | 2.0 | ± | 5.8 | 2.9 | ± | 7.8 | 1.1 | ± | 0.4 | 3.5 | ± | 8.7 | 1.0 | ± | 0.3 | 2.5 | ± | 6.8 | 0.663 | 0.129 | 0.612 |
| **arterial blood gas analysis** | |  |  |  |  |  |  |  |  |  |  |  |  |  |  |  |  |  |  |  |  |
| pO2 (mmHg) | 64.8 | ± | 14.4 | 67.6 | ± | 13.9 | 66.5 | ± | 14.1 | 64.0 | ± | 14.6 | 68.0 | ± | 19.7 | 64.7 | ± | 12.7 | 0.43 | 0.405 | 0.358 |
| pCO2 (mmHg) | 36.8 | ± | 8.4 | 37.7 | ± | 7.2 | 36.6 | ± | 9.3 | 37.4 | ± | 6.6 | 36.7 | ± | 12.1 | 37.1 | ± | 6.9 | 0.39 | 0.143 | 0.136 |
| AaDO2 (mmHg) | 32.7 | ± | 12.3 | 26.6 | ± | 15.2 | 32.6 | ± | 11.5 | 30.1 | ± | 14.9 | 34.0 | ± | 8.9 | 30.8 | ± | 13.9 | 0.062 | 0.362 | 0.373 |
| **right heart catheterisation** | | | |  |  |  |  |  |  |  |  |  |  |  |  |  |  |  |  |  |  |
| PAPm (mmHg) | 39.0 | ± | 15.7 | 42.0 | ± | 14.3 | 36.6 | ± | 12.7 | 43.2 | ± | 17.7 | 40.4 | ± | 14.8 | 39.4 | ± | 15.7 | 0.289 | **0.037** | 0.681 |
| RAPm (mmHg) | 10.9 | ± | 5.6 | 12.6 | ± | 4.5 | 9.9 | ± | 5.3 | 12.9 | ± | 5.1 | 10.0 | ± | 4.8 | 11.6 | ± | 5.5 | 0.107 | **0.002** | 0.289 |
| Cardiac index (L/min/m^2^) | 2.5 | ± | 0.7 | 2.2 | ± | 0.5 | 2.6 | ± | 0.8 | 2.2 | ± | 0.5 | 2.6 | ± | 0.7 | 2.4 | ± | 0.7 | 0.182 | **0.010** | 0.289 |
| PCWP (mmHG) | 15.4 | ± | 6.3 | 17.1 | ± | 6.1 | 14.7 | ± | 6.3 | 17.1 | ± | 6.0 | 15.8 | ± | 6.9 | 15.8 | ± | 6.1 | 0.354 | **0.045** | 0.835 |
| PVR (dynxsxcm-5) | 501.7 | ± | 317.8 | 555.2 | ± | 277 | 430.3 | ± | 269.5 | 621.9 | ± | 330.8 | 454.4 | ± | 302.2 | 527.4 | ± | 312.6 | 0.381 | **0.002** | 0.332 |
| SvO2 (%) | 64.7 | ± | 8.7 | 68.1 | ± | 11.5 | 66.8 | ± | 8.1 | 63.6 | ± | 10.5 | 66.2 | ± | 9.3 | 65.1 | ± | 9.4 | 0.173 | 0.104 | 0.637 |
| TPG (mmHG) | 22.7 | ± | 15.4 | 25.3 | ± | 11.5 | 21.5 | ± | 12.7 | 25.5 | ± | 16.7 | 24.6 | ± | 16.4 | 22.9 | ± | 14.3 | 0.123 | 0.128 | 0.964 |
| **echocardiography** |  |  |  |  |  |  |  |  |  |  |  |  |  |  |  |  |  |  |  |  |  |
| sPAP (mmHg) | 49.9 | ± | 18.3 | 59.7 | ± | 25.3 | 47.7 | ± | 17.6 | 57.1 | ± | 22.1 | 48.3 | ± | 19.7 | 52.9 | ± | 20.3 | 0.140 | 0.051 | 0.508 |
| TAPSE (mm) | 20.7 | ± | 7.2 | 25.5 | ± | 7.0 | 22.4 | ± | 6.5 | 21.2 | ± | 8.2 | 22.8 | ± | 7.9 | 21.6 | ± | 7.4 | 0.102 | 0.616 | 0.777 |
| RVEDD (mm) | 34.7 | ± | 9.7 | 34.5 | ± | 7.7 | 32.9 | ± | 7.9 | 36.5 | ± | 10.3 | 38.2 | ± | 6.1 | 34.1 | ± | 9.5 | 0.936 | 0.100 | 0.213 |
| LVEF (%) | 58.9 | ± | 9.3 | 57.3 | ± | 9.0 | 59.1 | ± | 9.1 | 57.9 | ± | 9.3 | 59.5 | ± | 12.1 | 58.4 | ± | 8.8 | 0.558 | 0.595 | 0.739 |
| **pulmonary function** |  |  |  |  |  |  |  |  |  |  |  |  |  |  |  |  |  |  |  |  |  |
| *DLCO (%)* | 65.1 | ± | 23.3 | 63.3 | ± | 25.1 | 67.4 | ± | 24.7 | 61.6 | ± | 22.4 | 81.4 | ± | 23.0 | 61.4 | ± | 22.5 | 0.799 | 0.363 | **0.018** |
| *KCO (%)* | 81.4 | ± | 27.4 | 74.6 | ± | 29.6 | 80.9 | ± | 26.6 | 78.9 | ± | 29.5 | 80.5 | ± | 25.1 | 79.9 | ± | 28.7 | 0.317 | 0.714 | 0.927 |

Data are represented as mean ± 1 standard deviation (SD); N depicts number of valid data for retrospective analysis; ID definitions: ID1, serum ferritin <30µg/L and TSAT <16%; ID2, serum ferritin <100µg/L and TSAT <20%; ID3, serum ferritin <100 µg/L or serum ferritin 100-299 µg/L and TSAT <20 %; abbreviations: RDW. red blood cell distribution width; MCV, mean corpuscular volume; MCH, mean corpuscular hemoglobin; NT-proBNP, N-terminal pro-B-type natriuretic peptide; CRP, C reactive protein; GFR, glomerular filtration rate; pO2, arterial partial pressure of oxygen; pCO2, arterial partial pressure of carbon dioxide; AaDO2, alveolar-arterial oxygen difference; PAPm, mean pulmonary arterial pressure; RAPm, mean right atrial pressure; PCWP, pulmonary capillary wedge pressure; PVR, pulmonary vascular resistance; SvO2, mixed venous saturation; TPG, transpulmonary pressure gradient (PAPm-PCWP); sPAP, systolic pulmonary arterial pressure; TAPSE, **tricuspid** annular plane systolic excursion; RVEDD, right ventricular end-diastolic diameter; LVEF, left ventricular ejection fraction; DLCO, diffusing capacity for carbon monoxide, depicted as percentage of normal; KCO, carbon monoxide transfer coefficient, also known as Krogh-Index (DLCO/VA, depicted as percentage of normal).
