# Supplementary material for: The crucial impact of iron deficiency definition for the course of precapillary pulmonary hypertension
Source: PLoS One. 2018 Aug 30;13(8):e0203396. doi: 10.1371/journal.pone.0203396 (PMC6117062; doi:10.1371/journal.pone.0203396)
Supplement: S7 Table — (DOCX) [file pone.0203396.s007.docx]

**S7 Table. Correlations of serum hepcidin with patients` characteristics at follow up in 2015.**

|  | **Spearman RHO`s correlation coefficient** |  |  | **Spearman RHO`s correlation coefficient** |
| --- | --- | --- | --- | --- |
| **clinical parameters** |  |  | **right heart catheterization** |  |
| age (years) | -0.252 |  | PAPm (mmHg) | -0.176 |
| BMI (kg/m^2^) | 0.152 |  | RAPm (mmHg) | -0.200 |
| SMWD (m) | -0.039 |  | Cardiac index (L/min/m^2^) | 0.235 |
| WHOFc | -0.236 |  | PCWP (mmHG) | -0.085 |
| Survival time | -0.205 |  | PVR (dynxsxcm-5) | -0.082 |
|  |  |  | SvO2 (%) | 0.085 |
| **laboratory parameters** |  |  | TPG (mmHG) | -0.194 |
| hemoglobin (g/l) | 0.247 |  |  |  |
| RDW (%) | **-0.436***** |  | **echocardiography** |  |
| MCV (fL) | 0.255 |  | sPAP (mmHg) | -0.084 |
| MCH (pg) | **0.406**** |  | TAPSE (mm) | -0.376 |
| serum iron (µmol/L) | -0.023 |  | RVEDD (mm) | -0.295 |
| transferrin (mg/dL) | **-0.401**** |  | LVEF (%) | 0.080 |
| transferrin saturation (%) | **0.414***** |  |  |  |
| ferritin (µg/L) | **0.565***** |  | **arterial blood gas analysis** |  |
| sTFR (mg/L) | **-0.419**** |  | pO2 (mmHg) | 0.007 |
| sTFRF index | **-0530***** |  | pCO2 (mmHg) | 0.183 |
| NTproBNP (ng/L) | -0.217 |  | AaDO2 (mmHg) | -0.024 |
| CRP (mg/dL) | 0.103 |  |  |  |
| GFR mL/min/1.73m^2^) | 0.026 |  | **pulmonary function tests** |  |
| uric acid (mg/dL) | 0.037 |  | DLCO (%) | 0.161 |
| creatinine (mg/dL) | -0.188 |  | KCO (%) | 0.046 |

Correlations were calculated with Spearman's Rho test and the correlation coefficient is shown. *p-value <0.05. **p-value<0.01. ***p-value <0.001. A significant correlation coefficient between +/- 0.1 to 0.3 was considered weak. from +/- 0.3 to 0.5 moderate and from +/- 0.5 to 1.0 strong; abbreviations: BMI, body mass index; RDW, red blood cell distribution width; MCV, mean corpuscular volume; MCH, mean corpuscular hemoglobin; NT-proBNP, N-terminal pro-B-type natriuretic peptide; CRP, C reactive protein; GFR, glomerular filtration rate; pO2, arterial partial pressure of oxygen; pCO2, arterial partial pressure of carbon dioxide; AaDO2, alveolar-arterial oxygen difference; DLCO, diffusing capacity for carbon monoxide; KCO, carbon monoxide transfer coefficient, also known as Krogh-Index (DLCO/VA); SMWD, six-minute walking distance; EHOFc, WHO functional class; PAPm; mean pulmonary arterial pressure; RAPm, mean right atrial pressure; PCWP, pulmonary capillary wedge pressure; PVR, pulmonary vascular resistance; SvO2, mixed venous saturation; TPG, transpulmonary pressure gradient (PAPm-PCWP); sPAP, systolic pulmonary arterial pressure; TAPSE, tricuspid annular plane systolic excursion; RVEDD, right ventricular end-diastolic diameter; LVEF, left ventricular ejection fraction.
