# Supplementary figures and images for: The crucial impact of iron deficiency definition for the course of precapillary pulmonary hypertension
Source: PLoS One. 2018 Aug 30;13(8):e0203396. doi: 10.1371/journal.pone.0203396 (PMC6117062; doi:10.1371/journal.pone.0203396)

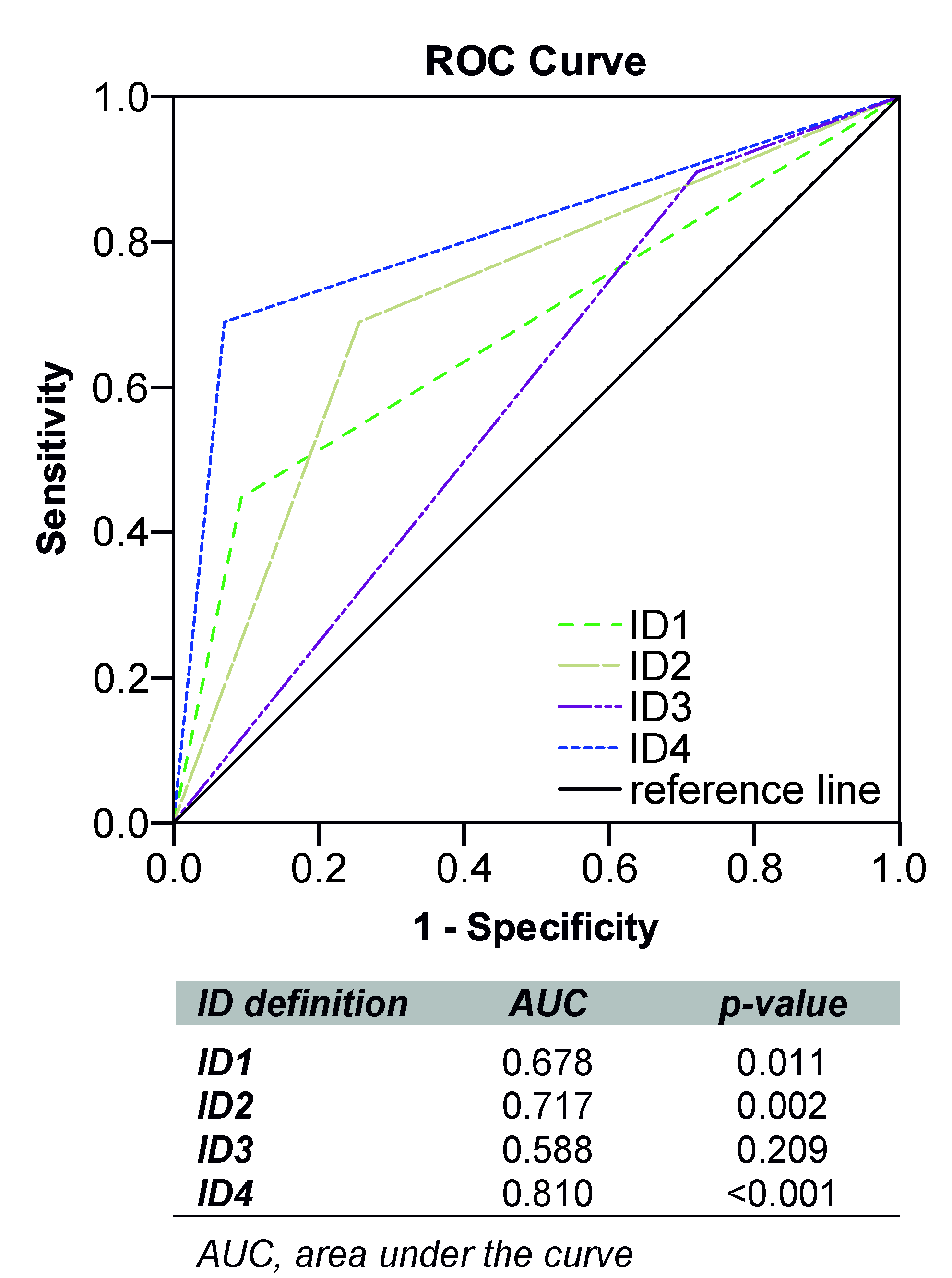

Supplement: S1 Fig — Detection of iron deficiency (ID) using soluble transferrin receptor/logferritin index (sTFRF index) was performed for pulmonary arterial hypertension patient evaluation in 2015. Other definitions for ID were compared to the sTFRF index based ID categorization (N = 103). The following definitions for ID were used: sTFRF index >3.2 if CRP <0.5 mg/dL or sTFRF index >2 if CRP >0.5 mg/dL (reference), ID1, serum ferritin <30μg/L and TSAT<16%; ID2, serum ferritin <100μg/L and TSAT <20%; ID3, serum ferritin <100μg/L or serum ferritin 100–299μg/L and TSAT<20%); ID4, sTFR>4.5 for women and >5.0 for men. (TIF) [file pone.0203396.s008.tif]
